# The Female Reproductive Microbiome: Mechanistic Insights and Bioengineering Perspectives

**DOI:** 10.3390/biology15161337

**Published:** 2026-08-07

**Authors:** María Belén Novoa Díaz, Pedro Carriere, Gabriel Vinderola, Claudia Gentili, Diego I Cattoni

**Affiliations:** 1 Department of Biology, Biochemistry and Pharmacy, National University of the South (UNS)-INBIOSUR (CONICET-UNS), Bahía Blanca 8000, Argentina; belen.novoa@uns.edu.ar (M.B.N.D.); pedro.carriere@uns.edu.ar (P.C.); 2 Institute of Industrial Lactology (INLAIN, CONICET-UNL), Faculty of Chemical Engineering, National University of the Littoral, Santa Fe 3000, Argentina; gvinde@fiq.unl.edu.ar; 3 Centre de Biologie Structurale (CBS), INSERM U1054, CNRS UMR5048, University of Montpellier, 34090 Montpellier, France; diego.cattoni@inserm.fr

**Keywords:** female fertility, reproductive microbiome, *Lactobacillus* spp., endometrial receptivity, vaginal dysbiosis, assisted reproductive technology (ART), synthetic biology, engineered microorganisms

## Abstract

Microbiota has emerged as a potential regulator of female reproductive health through immunological, metabolic, and endocrine networks. Growing evidence suggests that the composition and stability of the vaginal, uterine, and gut microbiota are associated with fertility outcomes. Disruptions in reproductive tract homeostasis have been linked to infertility, implantation failure, pregnancy loss, and diminished success in assisted reproductive technologies. Beyond local interactions, maternal gut microbiota may influence systemic immunity and metabolic pathways related to vaginal and endometrial microbiota. While these findings highlight the microbiome-based signatures’ potential as predictive and prognostic biomarkers, their clinical applicability remains unconfirmed. Evidence is limited by small cohort sizes, methodological and analytical heterogeneity, and lack of standardization, limiting clinical translation. This narrative review summarizes the current knowledge regarding the microbiome’s role in female reproductive health, highlighting its potential impact on pathophysiology, diagnostics, and therapeutic strategies. While this approach allows for a broad conceptual overview, we explicitly note that it is not systematic. As a result, this review is limited by the absence of a standardized search protocol, which may introduce selection bias. Finally, we review advances in microbial engineering and synthetic biology, highlighting engineered living biotherapeutics as promising strategies to improve microbiome-based reproductive medicine.

## Simple Summary

Female fertility depends not only on hormones and reproductive organs, but also on the communities of microorganisms that naturally live in the body (the microbiome). Recent research shows that bacteria present in the gut, vagina, and the more recently recognized communities in the uterus play an important role in regulating inflammation, immune balance, and the overall conditions required for successful pregnancy. Unbalance of these microbial communities is associated with fertility problems, including difficulty conceiving, repeated implantation failure during assisted reproduction, and early pregnancy loss. Scientists are increasingly exploring whether these microorganisms or their bioactive factors could be used as biomarkers to predict reproductive outcomes or to improve fertility treatments. However, current studies remain limited by differences in diagnostic methods and patient populations, making clinical application challenging. This review summarizes current knowledge about the relationship between the microbiome and female reproductive health, including findings from clinical studies and emerging therapeutic approaches such as probiotics. In addition, it explores future treatment strategies based on the development of bioengineered beneficial bacteria capable of improving the uterine environment and regulating inflammation. These advances may open new possibilities for personalized treatments in reproductive medicine.

## 1. Introduction

Developed nations are currently facing an unprecedented demographic challenge characterized by declining natality rates and a widespread trend toward delayed childbearing [1,2]. This shift has led to an escalating global reliance on assisted reproductive technologies (ART) [3]. Gynecological and pregnancy-related disorders continue to represent a major challenge for reproductive health, as the overall success rates remain limited by the complex physiological requirements of embryo implantation and early pregnancy [4,5]. Female fertility is a complex, multifactorial capacity that depends on the coordinated interaction of endocrine, metabolic, immunological, and environmental factors, together with the structural and functional integrity of the reproductive tract [4,6,7]. Disruption of these tightly regulated conditions may contribute to gynecological diseases and adverse reproductive outcomes, including infertility, implantation failure, and pregnancy complications [6,8,9].

In this context, the human microbiota has recently emerged as a potential regulator of female reproductive physiology with growing evidence that microbial communities influence fertility outcomes by modulating local and systemic immune, metabolic, endocrine, and inflammatory pathways [10]. Concomitantly, advances in microbial engineering and synthetic biology introduced new avenues for microbiome-based therapeutic interventions in reproductive medicine [11,12].

Female reproductive tract dysbiosis, commonly characterized by depletion of beneficial *Lactobacillus* species and enrichment of anaerobic taxa, has been associated with infertility-related events such as recurrent implantation failure, early pregnancy loss, and reduced success in ART [13,14]. Beyond local interactions, the gut microbiota may also influence reproductive function through distal effects (e.g., systemic dissemination of its metabolites or the putative dendritic-cell-mediated translocation of microorganisms). By shaping microbial communities in reproductive tissues, these events may contribute, directly or indirectly, to the development of female infertility disorders [15,16].

Recent advances further demonstrate that microbial composition and metabolic activity can be therapeutically modulated through lifestyle interventions, probiotics, microbial-derived metabolites, and fecal or vaginal microbiota transplantation approaches [17,18,19]. Accordingly, multiple preclinical and clinical studies have explored microbiome-targeted interventions in women with gynecological disorders and during pregnancy, highlighting the microbiota as both a potential biomarker and therapeutic target in reproductive medicine [17,20,21]. Despite these advances, current knowledge remains fragmented across different microbial niches, therapeutic approaches, and methodological platforms. Moreover, although microbial engineering and synthetic biology are rapidly expanding in other areas of medicine, their potential application to female reproductive health has yet to be fully explored. Therefore, this narrative review aims to summarize and discuss the current perspectives on the microbiome’s role in female reproductive health, with particular emphasis on fertility-associated microbial communities, microbiome-targeted therapeutic strategies, methodological challenges, and emerging bioengineering approaches. While this approach allows for a broad conceptual overview, we explicitly note that it is not systematic. As a result, this review is limited by the absence of a standardized search protocol, which may introduce selection bias. Additionally, this review integrates current knowledge with unresolved controversies, translational opportunities, and key knowledge gaps that should be addressed in future research.

## 2. The Reproductive Microbiome Axis

The human microbiome consists of complex communities of microorganisms that coexist in a physiological and symbiotic relationship with the host [22,23]. The female reproductive tract is shaped by age, ethnicity, lifestyle, and menstrual cycle, and is increasingly recognized as a highly interconnected microbial ecosystem in which the gut, vaginal, and endometrial microbiota establish a dynamic axis that profoundly impacts reproductive physiology and fertility outcomes [24,25].

The vaginal microbiota constitutes the first line of defense in the female reproductive tract and plays a critical role in maintaining gynecological health. In reproductive-age women, vaginal microbiota is generally classified into five community state types (CSTs). CSTs I, II, III, and V are predominantly composed of a single *Lactobacillus* species—*L. crispatus*, *L. gasseri*, *L. iners*, and *L. jensenii*, respectively—whereas CST IV is distinguished by a heterogeneous microbial community enriched in facultative and obligate anaerobic bacteria [26]. Ongoing research, however, reveals unprecedented diversity in the vaginal microbiome. This may challenge the established categories of dominance [26] in the near future. This classification is clinically relevant since different *Lactobacillus* taxa may exert distinct metabolic and immunomodulatory functions [17]. Healthy microbiota contributes to vaginal homeostasis through lactic acid production, maintenance of mucosal pH, barrier integrity, and inhibition of opportunistic pathogens. A differentiating element of the vaginal microbiota is that its composition undergoes dynamic temporal fluctuations linked to the menstrual cycle [25].

Beyond the vaginal compartment, the upper reproductive tract has long been considered a sterile environment [25,27]. Advances in next-generation sequencing (NGS) technologies for detecting bacterial 16S rRNA genes and metagenomic analyses have challenged this paradigm and revealed the existence of distinct microbial communities within the uterus [27,28], although with substantially lower bacterial biomass than in the vaginal environment (10^2^–10^4^-fold lower) [14,29]. Furthermore, accumulating evidence suggests that the endometrium harbors a *Lactobacillus*-dominated microbiota [14,27], particularly *L. iners*, *L. crispatus*, and *Prevotella* spp. [30,31]. Endometrial fluid and biopsy samples revealed a more complex composition that solely *Lactobacillus* spp., including *Anaerococcus*, *Atopobium*, *Bifidobacterium*, *Corynebacterium*, *Gardnerella*, *Haemophilus*, *Microbacterium*, *Prevotella*, *Propionibacterium*, *Staphylococcus*, and *Streptococcus* among others [12,30,31] and even a variety of other types of active microorganisms, such as fungi, viruses, and archaea [32]. Although it is widely accepted that the vast majority of these microorganisms ascend from the lower reproductive tract, growing evidence suggests a potential translocation of microbes from distant microbial communities [33,34]. In the debate on the presence of microorganisms in the uterine cavity, there is still no consensus regarding the existence of a stable resident endometrial microbiota or its characteristic taxonomic composition. The conflicting evidence is largely attributed to methodological and biological factors, including sample collection procedures, contamination, inter-study variability, and the cohort’s heterogeneity [14,27]. The existence of a placental microbiome remains more controversial and has been extensively debated in recent years [35]. De Goffau and colleagues reported no evidence of bacterial presence in the vast majority of placental samples from both uncomplicated and complicated pregnancies [36], and similar findings were subsequently reported by Panzer and colleagues [37]. In contrast, other studies have suggested that the placenta and amniotic fluid are not completely sterile environments and that microbial colonization may influence gestation, fetal development, and parturition [38,39]. Several authors have proposed that microorganisms from the vaginal, oral, or gut microbiota may translocate to the placenta and amniotic cavity, exerting either beneficial or detrimental effects on maternal–fetal physiology [4,33,40,41]. In this context, recent reviews have emphasized the methodological challenges of studying the placental and fetal membrane microbiota, particularly because traditional morphologic and culture-based approaches may lack sufficient sensitivity and reliability. Indeed, only a limited fraction of commensal microorganisms can be successfully cultured ex vivo, potentially leading to an underestimation of microbial diversity and abundance [33]. In contrast, high-throughput sequencing technologies have identified bacterial DNA signatures in umbilical cord blood, amniotic fluid, placenta, and fetal membranes [33,42]. These studies have described placental microbial profiles enriched in taxa belonging to *Firmicutes*, *Tenericutes*, *Proteobacteria*, *Bacteroidetes*, and *Fusobacteria* [33,43]. Satokari and collaborators have also detected bacterial DNA from *Bifidobacterium* and *Lactobacillus* species in placental samples, suggesting a potential role in maternal–fetal immune modulation [39]. Nevertheless, whether these findings reflect true placental colonization or contamination associated with low-biomass samples remains unresolved.

Historically, the intestinal microbiota has been studied more than the female reproductive microbiota, due to less invasive sampling techniques and the belief that the upper reproductive tract was a sterile environment [44]. Besides protozoa, viruses, and fungi, there are approximately 100 trillion bacteria in the gut, among which *Bacteroidetes* (*Prevotella*, *Porphyromonas*), *Firmicutes* (*Clostridium*, *Eubacteria*), *Proteobacteria*, and *Actinobacteria* (*Bifidobacteria*) stand out [4,45,46]. Gut microorganisms act as central regulators of systemic homeostasis since, in physiological conditions, limit pathogen colonization and proliferation, produce bioactive factors, influence endocrine metabolism, and modulate inflammatory and immune responses at both local and systemic levels [45,47,48,49]. In this context, the gut microbiota promotes immune cell differentiation and contributes to preserving intestinal barrier function, thereby limiting the systemic translocation of pathogenic bacteria and their pro-inflammatory products such as lipopolysaccharide (LPS) [50,51,52]. Regarding the active metabolites, the most studied are short-chain fatty acids (SCFAs) [50]. Among these, butyrate and propionate have attracted particular attention due to their anti-inflammatory properties, potentially influencing reproductive tract physiology and gestational success [47,53]. Gut microbiota have also been recognized as a component of an endocrine-like organ since it influences estrogen metabolism through the estrobolome, a collection of microbial genes encoding enzymes involved in estrogen deconjugation and recirculation. These processes directly impact ovarian steroidogenesis, endometrial receptivity, and embryo implantation [15]. Recent evidence suggests a dynamic crosstalk among the vaginal, endometrial, and intestinal microbial ecosystems through endocrine, metabolic, and immunological pathways [28]. This communication may occur through immune-cell-mediated bacterial trafficking, where dendritic cells and macrophages may transport microorganisms or their components from the gut to distant reproductive tissues, further contributing to the establishment and modulation of the vaginal, endometrial, and placental compartments [6,7,10,15,54].

**Figure 1:**
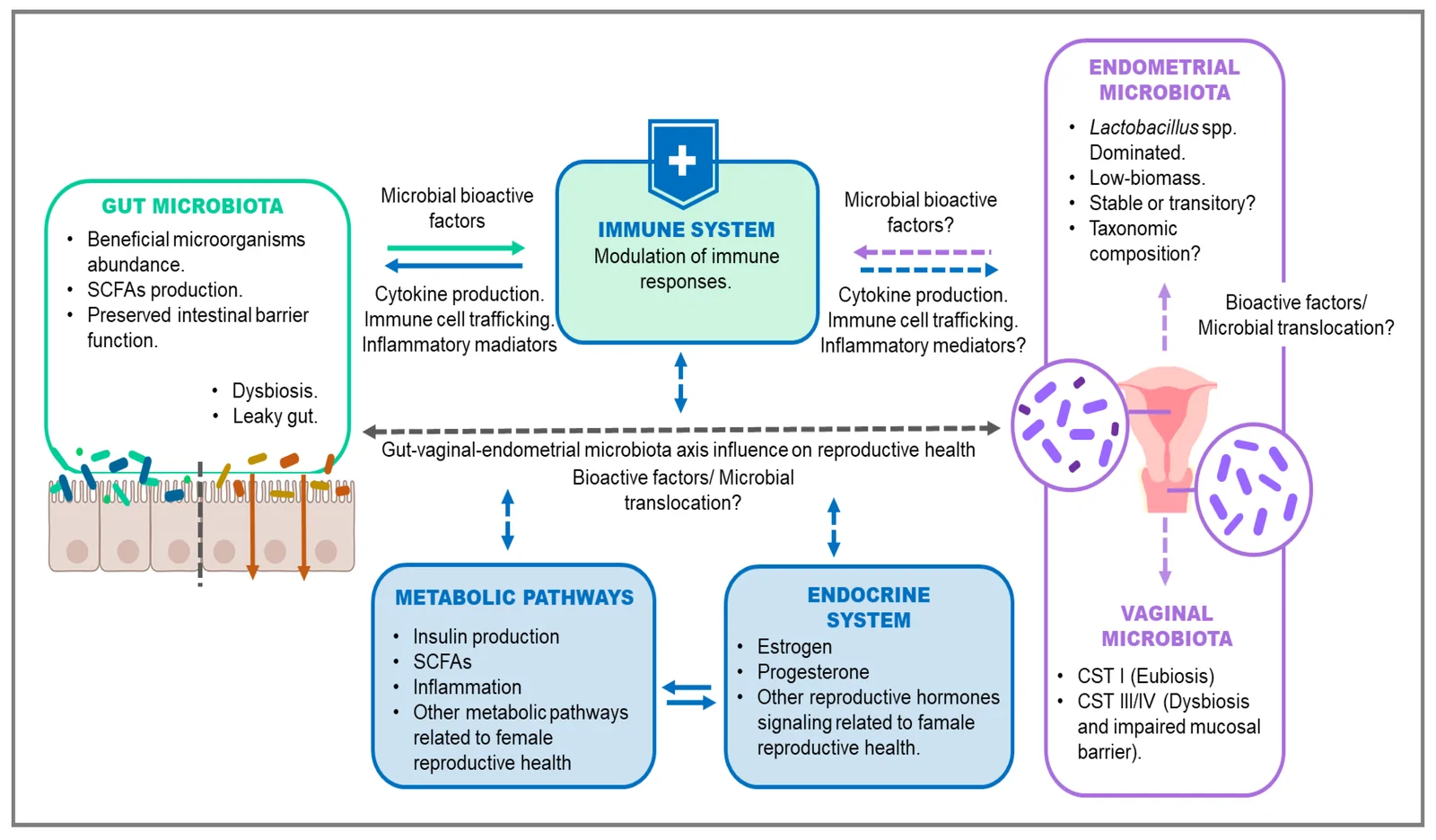
Proposed microbiota axis in female reproductive health. Conceptual model of the potential interactions linking the gut, vaginal, and endometrial microbiota with immune, metabolic, and endocrine pathways. Validation in well-designed human studies is still needed. The uterus illustration was adapted from a CC0 image available on Wikimedia Commons; the intestinal epithelium illustration was adapted from Barton JR et al., Wikimedia Commons (CC BY 4.0).

Clinically, disruption of this microbial network has been associated with multiple gynecological and obstetric disorders, including endometriosis, polyendocrine metabolic ovarian syndrome (PMOS, formerly polycystic ovary syndrome—PCOS), chronic endometritis, recurrent implantation failure, recurrent pregnancy loss, and major obstetric syndromes like preeclampsia [55,56,57,58]. Although microbial signatures vary considerably among studies, common hallmarks include reduced microbial diversity, depletion of beneficial SCFAs-producing bacteria, increased abundance of inflammatory taxa, and impaired mucosal barrier function [26]. However, despite growing evidence supporting the role of the reproductive microbiome axis in female fertility, major challenges remain, including methodological heterogeneity, low reproducibility among cohorts, contamination issues in low-biomass sequencing, and a lack of standardized diagnostic criteria. Overall, these findings position the reproductive microbiome axis as a potential regulator of female reproductive health and a promising future target for precision medicine approaches in reproductive biology. Further integration of metagenomics, metabolomics, immune profiling, and synthetic biology may enable personalized microbiome-based diagnostics and therapeutic interventions aimed at restoring reproductive tract homeostasis and improving fertility outcomes. The complex interplay among the female microbial reproductive axis, immune, metabolic, and endocrine pathways discussed throughout this section is summarized in Figure 1.

## 3. Microbial Dysbiosis and Reproductive Failure

The female reproductive tract microbiota plays a central role in endometrial receptivity, embryo implantation, immune tolerance, and pregnancy maintenance [26]. While a *Lactobacillus*-dominated vaginal and endometrial environment is generally associated with reproductive health, disruption of this microbial balance has increasingly been linked with adverse reproductive consequences [14,17,26]. Vaginal or endometrial microbiota shifts induced by infections with well-known microorganisms, including *Chlamydia trachomatis*, *Mycoplasma genitalium*, and *Neisseria gonorrhoeae*, result in subclinical alterations that are considered risk factors for poor reproductive outcomes [41]. Yet, the majority of cases of female genital dysbiosis are characterized by depletion of *Lactobacillus* spp. together with enrichment of anaerobic and potentially pathogenic microorganisms, including *Gardnerella*, *Atopobium*, *Prevotella*, *Sneathia*, *Streptococcus*, and *Mobiluncus* species [25,59]. These microbial profiles may promote epithelial barrier disruption, local inflammation, altered cytokine secretion, immune dysregulation, and impaired maternal–fetal tolerance. Also, they have been linked to impaired implantation, reduced pregnancy rates following ART, and inflammatory dysregulation in women with recurrent miscarriage [25,59,60]. In particular, Koedooder and colleagues demonstrate that women with low vaginal *Lactobacillus* abundance, particularly those enriched with *Gardnerella vaginalis* and *Atopobium vaginae*, had a lower probability of clinical pregnancy after ART. On the contrary, women with an abundance of *L. crispatus* had a high probability of pregnancy [59]. The mechanisms by which the vaginal and endometrial microbiota influence endometrial receptivity and embryo implantation are thought to be predominantly immunological. When microorganisms invade the vaginal or endometrial mucosa and activate pattern recognition receptors (PRRs) in epithelial and innate immune cells, a cascade of cytokines and chemokines is released, modulating the local immune microenvironment and shaping lymphocyte recruitment and activation. In contrast, *Lactobacillus* species contribute to mucosal homeostasis by inhibiting pathogen overgrowth through lactic acid production and by interacting with PRRs to promote immunoregulatory and anti-inflammatory responses [13,14,34]. Accumulating evidence indicates that intestinal dysbiosis may also contribute to reproductive dysfunction through endocrine, metabolic, and immunological mechanisms [61]. Over the past decade, this concept has been reinforced by the growing number of studies linking gut microbial dysbiosis to a wide spectrum of gynecological and pregnancy-related disorders [20,21,62,63]. Tersigni and collaborators investigated the relationship between intestinal permeability and recurrent pregnancy loss in a cohort of 70 affected women and 30 healthy controls, showing a statistically significant association between impaired intestinal barrier integrity, elevated inflammatory parameters, and recurrent pregnancy loss [64]. Mechanistically, increased intestinal permeability may promote systemic translocation of LPS and circulating immune cells that subsequently migrate to vaginal or endometrial tissues, thereby contributing to a chronic pro-inflammatory environment [65,66]. In particular, increased endometrium infiltration of type 1 T helper cells (Th1) and Th17 lymphocyte populations, together with reduced numbers of regulatory T cells (Treg) and tolerogenic natural killer cells (NK), have been strongly associated with a pro-inflammatory environment, impaired maternal–fetal immune tolerance, and defective embryo implantation [63]. Taken together, female reproductive or gestational disorders are increasingly understood within the context of a bidirectional gut–vagina–endometrium microbiota axis in which microbial bioactive factors, immune-cell trafficking, inflammatory mediators, and hormonal signaling interact across distant mucosal sites (see Figure 1 and Table 1 and Table 2). In the following paragraphs, we examine how dysbiosis of this axis has been implicated in four representative conditions: endometriosis (i), PMOS (ii), chronic endometritis (iii), and pregnancy-related complications such as preeclampsia (iv).

(i) Endometriosis is a chronic inflammatory disorder characterized by the ectopic growth of endometrial-like tissue outside the uterine cavity, which impairs fertility and reproductive outcomes. Its development and progression are strongly influenced by estrogen signaling; however, in vivo studies have demonstrated that endometriotic lesions may persist even after ovariectomy, suggesting that additional mechanisms contribute to disease establishment and maintenance [15]. In recent years, increasing evidence has highlighted the potential involvement of the microbiota in endometriosis pathophysiology. Several investigations have reported vaginal, endometrial, and gut microbial alterations in women with endometriosis. Although reduced *Lactobacillus* spp. abundance has been consistently reported in women with endometriosis [7,67,68], the specific bacterial taxa associated with dysbiosis vary substantially across studies. In reproductive tract samples, Hernandes and colleagues identified increased abundance of *Alishewanella*, *Enterococcus*, and *Pseudomonas* [68], whereas studies evaluating the gut microbiota have identified distinct microbial alterations, including enrichment of *Sneathia*, *Barnesiella*, and *Gardnerella* [67], or increased abundance of *Bacteroides* and *Parabacteroides* [69].
(ii) PMOS is a common endocrine and metabolic disorder among women of reproductive age and has also been associated with microbial imbalance. In the lower reproductive tract of affected women with PMOS, decreased *Lactobacillus* spp. and increased prevalence of *Gardnerella vaginalis*, *Prevotella*, and *Mycoplasma* species have been reported [70,71]. In addition, several investigations suggest the existence of an altered endometrial microbial composition [72,73], while intestinal dysbiosis has also been associated with PMOS [74,75]. Women with PMOS exhibit reduced gut microbial diversity, increased intestinal permeability, chronic low-grade inflammation, and metabolic dysfunction. Zhou and colleagues demonstrated markedly decreased gut microbial diversity in obese women with PMOS, together with enrichment of *Lachnoclostridium* and *Fusobacterium* [75]. Likewise, Lindheim and coworkers identified significant alterations in gut microbial composition associated with hyperandrogenism and insulin resistance in PMOS patients [76]. Although most studies agree on reproductive tract and gut dysbiosis, the specific bacterial taxa associated with the disorder differ considerably among cohorts. These discrepancies may reflect differences in obesity status, insulin resistance, hyperandrogenism, ethnicity, dietary habits, and sequencing methodologies, making it difficult to define a universal microbial signature for PMOS.
(iii) Chronic endometriosis and PMOS are characterized by persistent low-grade inflammation of the endometrial mucosa and have been strongly associated with infertility and recurrence in both implantation failure and pregnancy loss [18]. Fewer studies have characterized the endometrial microbiota in chronic endometritis, consistent with findings in other reproductive disorders. Depletion of *Lactobacillus* spp. and increased abundance of opportunistic pathogens appear to be the hallmark of the reproductive tract dysbiosis associated with this condition. In fact, Wang and colleagues reported a decrease in *Lactobacillus* and an increase in opportunistic pathogens, including *Gardnerella* and *Sphingomonas*, along with a higher prevalence of *Enterococcus* and *Ureaplasma* in patients with this disease [77]. However, other studies have identified distinct microbial profiles, including enrichment of *Proteobacteria*, *Aminicenantales*, and *Chloroflexaceaein* chronic endometritis, while *Lactobacillus*, *Acinetobacter*, *Herbaspirillum*, *Ralstonia*, *Shewanella*, and Micrococcaceae were related to non-endometriotic patients [78]. The inconsistency across studies highlights the need for larger and standardized cohorts. Still, most investigations describe loss of *Lactobacillus* spp. dominance as the principal feature of chronic endometritis-associated dysbiosis, suggesting that alterations in the overall microbial community may be more reproducible than changes in individual taxa. Regarding gut dysbiosis, although human evidence linking these shifts directly to chronic endometritis remains limited, animal studies strongly support a mechanistic association between intestinal microbial imbalance, systemic inflammation, and the development of endometrial inflammation [79,80,81].
(iv) Preeclampsia is a pregnancy-specific hypertensive disorder associated with maternal and fetal complications. Although direct evidence linking endometrial microbiota alterations with preeclampsia remains scarce, several studies have reported significant associations between vaginal dysbiosis and gestational complications. Anaerobic bacteria, in particular, may promote a pro-inflammatory environment that contributes to the pathophysiology of preeclampsia. Lin and colleagues found that enrichment of *Prevotella bivia* in vaginal microbiota may be involved in its development [82]. Because the gut microbiota and its metabolites are closely linked to placental function and development [19,83], intestinal dysbiosis has likewise been associated with preeclampsia or eclampsia [84,85]. Notably, a significant reduction in gut microbial diversity has consistently been observed in affected women compared with healthy pregnant controls [86]. However, the microbial signatures associated with this disorder remain controversial. Some investigations have identified intestinal overgrowth of the genus *Blautia* as a potential risk factor [87,88], whereas other studies have reported the opposite association [89]. Jin and collaborators analyzed fecal samples from 92 women with preeclampsia and 86 healthy pregnant women, revealing significant alterations in microbiota diversity in affected patients. These changes were characterized by enrichment of potentially harmful bacteria and depletion of SCFAs-producing microorganisms. Moreover, the authors proposed a reduced abundance of *Akkermansia muciniphila* as a potential diagnostic biomarker for preeclampsia [21].

Conflicting findings have also been reported regarding the role of *Bifidobacterium* in preeclampsia. Altemani and colleagues observed increased abundance of this genus in the gut microbiota of preeclamptic patients [90]. In contrast, several other studies have suggested that *Bifidobacterium* may exert protective effects against the development of this disorder [84,85,87]. Consistently, a recent Mendelian randomization analysis based on data from 5731 women with preeclampsia/eclampsia and 160,670 controls, demonstrated a statistically significant association between higher maternal intestinal abundance of *Bifidobacterium* and reduced risk of developing this syndrome [91]. These apparently conflicting findings may reflect differences in study populations, ethnicity, dietary habits, gestational age at sampling, disease severity, sample type, sequencing platforms, and bioinformatic pipelines, all of which are known to influence microbiome profiling. Moreover, because *Bifidobacterium* comprises multiple species with distinct metabolic and immunomodulatory properties, genus-level analyses could mask species or strain-specific associations with preeclampsia. All these contradictory observations illustrate the complexity of defining disease-specific microbial biomarkers in preeclampsia, as well as in other fertility-associated pathologies, and suggest that microbial function and metabolite production may be more informative than taxonomic composition alone. Although the precise mechanisms underlying all these reproductive disorders remain incompletely understood, the evidence discussed throughout this section strongly suggests that alterations in microbiota composition and metabolic activity may contribute to their onset and progression. Table 1 and Table 2 summarize the most relevant alterations in the vaginal/endometrial and gut microbiota, respectively, associated with infertility and reproductive disorders, together with the proposed molecular and immunological mechanisms involved. Collectively, these findings reinforce the concept of the gut–reproductive axis as a critical regulator of female reproductive health and a promising target for future diagnostic and therapeutic strategies.

**Table 1:** Vaginal and endometrial alterations associated with infertility and related disorders. Summary of the principal alterations described in the vaginal and endometrial microbiota associated with infertility and reproductive disorder pathologies, including their proposed molecular and immunological mechanisms.

Microorganisms	Proposed Mechanisms	Reproductive Outcome/Associated Disorder	References
*L. crispatus*	Lactic acid production; bacteriocin secretion; TLR inhibition; reduced pro-inflammatory cytokines.	Mucosal milieu permissive for embryo attachment. Favors trophoblast invasiveness and decidual macrophages differentiation.	[13,14,34,59,92,93,94]
*L. iners*	Limited metabolic capacity; reduced acidification.	Induces microbiome states that are prone to dysbiosis. It is associated with relapse following standard antibiotic therapy for bacterial vaginosis.	[17,95,96]
*E. coli*, *Streptococcus*, *Shigels*, *Proteus*, *Enterobacteria*, and *Candida* spp.	Pro-inflammatory mediators; placental/fetal membrane colonization; sepsis.	Negative impact on pregnancy rates. Premature rupture of membranes, preterm labor, and clinical and subclinical chorioamnionitis.	[41]
Decrement of *Lactobacillus* spp. Increment of *Gardnerella*, *Atopobium*, *Prevotella*, *Sneathia*, *Streptococcus*, and *Mobiluncus* spp.	Barrier dysfunction; local inflammation; immune dysregulation; impaired maternal–fetal tolerance.	Affect implantation, placentation, and pregnancy progression.	[14,61]
Dysbiotic communities	Altered NK and macrophage polarization; Th1/Th2 imbalance; reduced Treg responses.	Pro-inflammatory signature, implantation failure, and pregnancy loss.	[63,97]
*Chlamydia trachomatis*, *Mycoplasma tuberculosis*, and *Neisseria gonorrhoeae*	Favor microbiota dysbiosis; subclinical inflammation; immune alterations.	Compromised reproductive environment. Impaired fertility and poor reproductive outcomes.	[41]
*Gardnerella* spp.	Biofilm formation; sialidase/toxin production; mucin remodeling; polymicrobial persistence.	Induce pro-inflammatory responses, impaired implantation.	[17]
Decreased *Lactobacillus* spp. abundance and increased prevalence of *Gardnerella vaginalis*, *Prevotella*, and *Mycoplasma* species	Reduced lactic acid; elevated pH; pathogen overgrowth. Dysbiosis alters hormone receptors, driving hyperandrogenism.	PMOS-associated infertility (ovulatory dysfunction, endocrine and metabolic disturbances, impaired endometrial receptivity, and chronic low-grade inflammation).	[71,73]
Decrease in *Lactobacillus*; enrichment of *Gardnerella*, *Atopobium*, *Escherichia/Shigella*, *Streptococcus*, *Anaerococcus.*	Reduced lactic acid; pathogen overgrowth; PRR/TLR–NF-κB activation; barrier dysfunction; Th1/Th17 polarization; reduced Treg responses.	Chronic inflammation and altered endometrial receptivity.	[7,67,68]
Enrichment of anaerobic and biofilm-forming taxa	Pro-inflammatory cytokine production (IL-1β, IL-6, TNF-α, IL-17).	Local inflammatory microenvironment associated with endometriosis progression.	[68,98]
*Prevotella*, *Gardnerella*, *Atopobium*	LPS/TLR activation; pro-inflammatory cytokine production.	Favor implantation failure, pathological inflammation, and adverse obstetric outcomes.	[17,99]
Decrease in *Lactobacillus* and higher prevalence of *Gardnerella*, *Sphingomonas*, *Enterococcus*, *Ureaplasma*, and *Prevotella*.	Proteolysis; neutrophil/Th17 activation; impaired endometrial remodeling.	Endometritis, impaired implantation.	[17,77,78]

Abbreviations: IL, interleukine; NF-κB, Nuclear factor kappa-light-chain-enhancer of activated B cells; Th, T helper cell; TNF-α, Tumor Necrosis Factor alpha; Treg, Regulatory T cell.

**Table 2:** Gut microbiota alterations associated with infertility and reproductive disorders. Summary of the principal alterations described in the gut microbiota associated with infertility and reproductive disorders, including their proposed molecular, metabolic, and immunological mechanisms.

Microorganisms	Proposed Mechanisms	Reproductive Outcome/Associated Disorder	References
Dysbiotic microbiota associated with decreased *Faecalibacterium*, *Bifidobacterium*, and *Akkermansia*	Leaky gut; LPS translocation; systemic immune cell trafficking; chronic inflammation.	Impaired maternal–fetal immune tolerance and defective embryo implantation.	[63,64,65,66]
*Lactobacillus* spp. and *Bifidobacteria* spp.	SCFAs production (particularly butyrate). Translocation of beneficial microorganisms by dendritic cells.	Influence epithelial cell biology and immune tone in the female reproductive tract, protecting the fetus from activated maternal immune cells	[33,41]
Enrichment of *Lachnoclostridium* and *Fusobacterium*	Reduced gut diversity and leaky gut. LPS leakage and chronic inflammation. Insulin resistance and increased androgen production.	PMOS-associated infertility (ovulatory dysfunction, endocrine and metabolic disturbances, impaired endometrial receptivity, and chronic low-grade inflammation)	[75,76]
Altered abundance of *Sneathia*, *Barnesiella*, and *Gardnerella* in stools. Enrichment of *Bacteroides* and Parabacteroides in the intestinal microbiota.	Increased intestinal permeability; systemic endotoxemia; altered estrobolome; reduced anti-inflammatory metabolites.	Chronic low-grade systemic inflammation associated with endometriosis progression.	[67,69]
Altered abundance of *Bifidobacterium*, *Akkermansia muciniphila*, *Blautia* and/or alterations in microbiota diversity.	Enrichment of potentially harmful bacteria and depletion of SCFAs-producing microorganisms.	Alterations associated with preeclampsia.	[21,87,88,89,91]

Abbreviations: LPS, lipopolysaccharide; PMOS, Polyendocrine Metabolic Ovarian Syndrome; SCFAs, Short-Chain Fatty Acids.

## 4. Biomarkers and Clinical Evidence Targeting the Female Fertility Microbiome

The marked global decline in fertility rates has become a major global public health concern [100]. Accumulating evidence indicates that the female reproductive tract and gut microbiota actively participate in local and systemic immune regulation, epithelial homeostasis, and endometrial receptivity. Thus, research studies have increasingly focused on translating microbiome profiling into clinically applicable biomarkers for infertility diagnosis, microbiome-based risk classification, and prediction of reproductive outcomes.

Recent meta-analyses show that women harboring a favorable vaginal and endometrial microbiome, particularly enriched in *Lactobacillus*, exhibit significantly higher implantation, pregnancy, and live birth rates than those with a dysbiotic microbiome [101,102], and, notably, denser and more interconnected microbial networks [102]. Conversely, reduced *Lactobacillus* spp. abundance and enrichment of anaerobic or pathogenic taxa are associated with a higher incidence of recurrent implantation failure, miscarriage, and chronic endometrial inflammation [101,103]. Consistently, CST III and IV microbiota, characterized by diminished *Lactobacillus* spp. predominance, have been linked to an increased risk of pregnancy loss [104].

Emerging evidence suggests that not all *Lactobacillus*-dominated communities confer equivalent reproductive benefits. While *L. crispatus* appears consistently associated with reproductive tract homeostasis and reproductive success [101,102], microbiota dominated by *L. iners* or *L. gasseri* may exhibit less stable ecological behavior and poorer ART outcomes [105]. These findings highlight the importance of moving beyond simple taxonomic abundance toward functional and ecological characterization of microbial communities.

Prospective clinical studies reinforce this translational potential. In a cohort of 342 infertile, infection-asymptomatic patients undergoing ART, endometrial fluid and biopsy sampling before embryo transfer showed that endometrial microbiota composition was associated with implantation success [14]. A comparable balance between *Lactobacillus* and pathogenic populations across both the endometrial and vaginal compartments has likewise been linked to ART success rates [13].

Complementary strategies have also emerged in the study of the gut microbiota and female reproductive disorders. Zhang and collaborators reported potential causal associations between specific intestinal taxa and gynecological pathologies [106]. The authors found that the *Eubacterium hallii* species exerted a protective effect against premature ovarian insufficiency while contributing to the development of endometriosis. In addition, the genus *Erysipelatoclostridium* was associated with both PMOS and endometriosis, whereas the Clostridiaceae family appeared to confer protection against uterine polyps. Reverse-direction analyses further suggested that endometriosis itself reduces *Bifidobacterium* spp. abundance [106]. Because the genetic instruments for microbial taxa classification are relatively weak, these causal estimates require replication. Even so, these findings suggest the concept of a gut–reproductive tract axis (Figure 1), and raise the possibility that intestinal microbial signatures may serve not only as biomarkers of disease susceptibility but also as therapeutic targets in reproductive medicine.

Moreover, recent studies suggest that microbiome-based biomarkers should not rely exclusively on taxonomic composition but rather integrate functional, immunological, and metabolic information. Liu and colleagues proposed a multi-omics framework integrating microbiome composition with transcriptomic, metabolomic, immunologic, and embryonic datasets to generate mechanistically informed biomarkers for precision reproductive medicine [107]. This work suggests that functional microbial activity and host–microbiome interactions may ultimately provide greater predictive value than taxonomic composition alone.

Despite all these findings, the clinical application of microbiome-based biomarkers is not yet sufficiently established. Diagnostic accuracy varies considerably across studies, whereas sensitivity, specificity, and predictive capacity are often difficult to compare due to differences in populations, samples, methodologies, and workflows. Furthermore, most of the proposed microbial signatures have not yet been validated in large, independent cohorts, which limits their reproducibility. Future clinical validation of microbiome-based biomarkers will require prospective multicenter studies including independent external validation cohorts and the harmonization of clinical endpoints to determine whether microbial signatures remain reproducible across different populations and clinical settings. Also, practical considerations should be addressed before clinical implementation, as current microbiome profiling approaches remain relatively expensive and require specialized laboratory infrastructure and bioinformatic expertise. Demonstration of clinical utility and cost-effectiveness will be critical before widespread implementation of microbiome-based biomarkers for diagnostic and prognostic applications in female reproductive health.

## 5. Current Limitations and Barriers to Clinical Translation of Microbiome Research

Despite promising observations, the clinical translation of microbiome-based diagnostics remains challenging for physiological and methodological reasons. Regarding patient physiology, the female microbiota varies significantly with factors such as age, ethnicity, diet, and hormonal status, limiting the extrapolation of findings across cohorts that have not controlled for these parameters [108]. For instance, changes in estrogen and progesterone levels across the menstrual cycle and pregnancy influence microbial stability and community structure within the gut and reproductive tract microbiomes [108]. The challenge is even greater for women undergoing hormonal treatments for infertility. It is well-documented that estrogen has a bidirectional regulatory link between the immune system and the gut, vaginal, and endometrial microbiota [101,109]. Zhao and coworkers demonstrated that women undergoing in vitro fertilization exhibited significantly reduced diversity and richness within the vaginal microbiome [110]. Consistent with these findings, Carosso and collaborators reported that ovarian stimulation and progesterone supplementation were associated with modest reductions in *Lactobacillus* spp. abundance and increased bacterial diversity in both the vaginal and endometrial microbiota. These alterations were accompanied by a relative increase in potentially pathogenic taxa, including *Prevotella* spp., *Escherichia coli*, *Shigella* spp., and *Atopobium* spp., suggesting that microbiome instability during ART may negatively affect endometrial receptivity and placentation [111]. Similarly, a longitudinal study monitoring consecutive intrauterine insemination and in vitro fertilization cycles identified progressive reductions in *Lactobacillus* spp. dominance, potentially increasing the risk of bacterial vaginosis and adversely impacting ongoing pregnancy rates [104].

From the technical standpoint, the limitations arise from sensitivity, specificity, and economic cost to deploy massive microbiota quantification technologies. The most widely used methodologies currently include culture-based techniques (culturomics), polymerase chain reaction (PCR)-dependent molecular methods, and NGS platforms [102]. Culturomics-based approaches combine the extensive cultivation of microorganisms under multiple culture conditions with subsequent identification by MALDI-TOF (Matrix-Assisted Laser Desorption/Ionization Time-of-Flight) mass spectrometry [34,112], enabling microbial identification at both genus and species levels through species-specific protein profiles, including low-abundance bacteria. In addition, it allows the recovery of viable microorganisms for downstream functional studies, such as antimicrobial susceptibility testing and host–microbiota interaction analyses. However, culturomics requires highly specific culture conditions, prolonged incubation periods, and extensive technical workload, substantially increasing operational costs and limiting its large-scale application [102,112,113]. Furthermore, many bacterial species inhabiting the female reproductive microbiome are difficult or impossible to culture under standard laboratory conditions, potentially leading to an underestimation of the genuine microbial diversity [114].

In contrast, PCR-based methodologies represent a faster, highly sensitive, and more cost-effective strategy for the detection and quantification of specific microorganisms associated with eubiosis and dysbiosis within the gut, vaginal, and endometrial microbiota [114,115]. These techniques enable the rapid detection of clinically relevant bacteria, even in low-biomass samples, making them attractive tools for both clinical practice and large-scale studies. Nevertheless, these methods constitute targeted approaches, as they require prior knowledge of the bacterial sequences of interest for the design of specific primers. Consequently, they do not allow comprehensive characterization of complex microbial communities or the detection of unexpected or previously undescribed microorganisms [102].

In recent years, NGS technologies have become a standard for the study of the human microbiota, enabling culture-independent microbial characterization even in samples with extremely limited biological material. The most commonly employed approach is 16S rRNA gene sequencing, which is used to characterize bacterial composition and estimate microbial diversity and relative abundance [114]. Among this type of technique, EMMA^®^ (Endometrial Microbiome Metagenomic Analysis, Igenomix) is one of the most widely used approaches to characterize endometrial bacterial composition. Although it profiles the entire bacterial composition, its clinical interpretation primarily relies on the relative abundance of *Lactobacillus* spp., classifying samples based on dominance. This analysis is frequently combined with ALICE^®^ (Analysis of Infectious Chronic Endometritis), which is designed to detect microorganisms associated with chronic endometritis. These platforms may provide clinically relevant microbiome profiling in patients with recurrent implantation failure, recurrent pregnancy loss, or unexplained infertility. However, their application remains limited by high costs, the requirement for specialized bioinformatic analysis, lack of standardized protocols for low-biomass samples, and the persistent risk of environmental or reagent-derived contamination (also known as “kitome contamination”) [14,107]. An additional challenge concerning this type of technology arises from the compositional nature of data obtained through microbiome sequencing. Since sequencing reveals relative rather than absolute abundances, an apparent increase in one bacterial species might simply reflect a decrease in another, rather than a true biological change. Consequently, it is essential to employ appropriate compositional data analysis methods to avoid or minimize biases in the interpretation of results.

Recent studies have incorporated shotgun metagenomics, metabolomics, and transcriptomics to obtain strain-level resolution and deeper functional characterization of microbial communities, enabling the evaluation of metabolic pathways, virulence-associated genes, and antimicrobial resistance determinants [108,116]. These approaches have revealed that microbial metabolic products and enzymatic functions may offer greater predictive value for health outcomes than taxonomic composition alone, thereby challenging traditional concepts of “beneficial” or “pathogenic” microbial profiles [108]. Nevertheless, the success of these culture-independent methodologies largely depends on the complexity of computational analyses and their interpretations, as well as on the quality and quantity of metagenomic DNA extracted from biological samples [114,116]. Thus, the isolation of high-quality microbial DNA from vaginal, endometrial, or fecal samples remains technically challenging. Factors including inefficient microbial lysis, host DNA contamination, sample handling, storage conditions, and DNA degradation may significantly affect sequencing performance and downstream bioinformatic analyses [102,114,117].

To evaluate the predictive value of these technologies, it is also essential to consider the limitations associated with sample type and collection procedures. Fecal samples are the most commonly used specimens for intestinal microbiota analysis due to their accessibility and non-invasive collection. However, whether fecal microbiota accurately reflects the full diversity and spatial heterogeneity of microbial communities inhabiting different regions of the gastrointestinal tract remains a matter of debate [118,119,120,121].

For lower and upper female genital tract microbiota analysis, vaginal sampling is a minimally invasive procedure and, owing to the high microbial load in this niche, carries a low risk of contamination [122]. For endometrial microbiota assessment, samples may be obtained either through endometrial biopsies, which require invasive procedures, or through less invasive catheter-based aspiration of endometrial fluid. Although the latter is technically simpler and more suitable for clinical practice, it carries a higher risk of contamination from vaginal and cervical microbiota during sample collection [25,122]. To overcome these limitations, several techniques and sampling devices have been developed over the last decade, enabling the aspiration or suction of endometrial fluid as well as brushing of the endometrial wall while minimizing contact with the cervical canal and vaginal walls [122]. Moreover, several authors have proposed longitudinal sampling strategies involving repeated collection from the same individuals across menstrual cycles or consecutive ART cycles, thereby improving the robustness, reproducibility, and biological representativeness of microbiome analyses [102,122]. In addition, strict protocols are required for low-biomass specimens such as placental tissue, fetal membranes, amniotic fluid, and endometrial samples, where microbial DNA is often close to the analytical detection limit. Current recommendations, therefore, emphasize rigorous contamination-aware workflows, including the use of multiple negative controls during sampling, DNA extraction, and sequencing; processing samples under sterile conditions; the use of decontamination algorithms; and complementary validation using culture-independent and targeted molecular approaches.

One strategy to mitigate the limitations of single-method, single-compartment analysis is the integration of -omics data across the clinically accessible reproductive compartments, including the follicular unit, endometrium, and embryo [107]. Each compartment provides distinct molecular signatures: transcriptomic and metabolic signatures associated with oocyte and follicular quality, molecular programs governing endometrial receptivity, and secretomic profiles released by developing embryos. Integrating these datasets through advanced computational analyses and systems biology modeling enables mechanistic patient stratification and can reveal coordinated dysfunctions that often remain undetectable using single-omics approaches alone [17,107].

The clinical deployment of these technologies is still in its early stages. Variability in methodological workflows, such as taxonomic classification algorithms, reference databases, normalization procedures, and statistical analyses, may lead to different biological interpretations even when the analyzed databases are identical. These differences contribute to the limited reproducibility observed across microbiome research and highlight the need of standardized analytical procedures. Such methodological limitations also affect the interpretation of commercially available microbiome-based diagnostic platforms. Although assays such as EMMA^®^ and ALICE^®^ have been increasingly incorporated into reproductive medicine, their clinical utilities remain under active investigation. Current evidence is insufficient to establish universally accepted thresholds that distinguish clinically relevant dysbiosis from physiological microbial variation [123] or to determine whether microbiome-directed interventions based on these profiles consistently improve reproductive outcomes. Most available studies have been conducted in selected infertile populations, frequently involving patients undergoing assisted reproductive technologies, which may limit the generalizability of findings to broader clinical settings.

## 6. Therapeutic Modulation of the Female Reproductive Microbiome

Because alterations in the vaginal, endometrial, and gut microbiota exert profound effects on host metabolism, immunity, and reproductive physiology, these ecosystems have emerged as a promising target for therapeutic intervention in infertility and gestational disorders [124,125]. Moreover, given the gut–vagina–endometrium axis, dietary modulation of gut microbiota, for example, through diet, may indirectly influence vaginal and endometrial microbial composition as well. Among these approaches, lifestyle interventions such as smoking cessation, reduced alcohol consumption, stress reduction, and regular physical activity have been associated with beneficial shifts in microbial diversity, immune balance, and metabolic health [126]. In parallel, nutritional interventions have gained particular attention as modulators of intestinal and reproductive tract microbiota. Prebiotics are defined by the International Scientific Association for Probiotics and Prebiotics (ISAPP) as a substrate that is selectively utilized by host microorganisms, conferring a health benefit. Prebiotics may promote the production of SCFAs and other microbial metabolites involved in immune modulation, epithelial integrity, and hormonal regulation [127,128]. Diets rich in fiber, prebiotics, and polyphenols, as well as fermented foods, have been associated with increased microbial diversity and enhanced abundance of beneficial taxa, particularly *Lactobacillus* and *Bifidobacterium* species [124,125].

Probiotics are defined as live microorganisms that, when administered in adequate amounts, confer health benefits to the host [129]. These beneficial microorganisms can modulate microbial diversity and abundance, alter microbial metabolic activity and metabolite production, enhance mucus secretion, reinforce epithelial barrier integrity, and exert broad immunomodulatory effects. Furthermore, through the production of SCFAs, probiotics are capable of influencing hormone metabolism, including insulin, androgen, and estrogen signaling [16,130]. Probiotics intended for the treatment of reproductive disorders, particularly during pregnancy, should fulfill several essential criteria. First, due to safety and ethical considerations, strains derived from the human microbiota have to be prioritized for clinical application. Second, they should be capable of temporarily colonizing the reproductive tract or the intestine without inducing inflammatory responses; in the case of vaginal probiotics, they should ideally promote efficient lactic acid production. Third, they should possess mechanisms capable of inhibiting pathogenic microorganism proliferation. Fourth, they should neither harbor nor transfer antibiotic resistance genes. Finally, their beneficial effects should be supported by clinical evidence demonstrating safety and efficacy [11,17].

Although the precise mechanisms through which probiotics influence female reproductive function remain largely unknown, increasing evidence from preclinical and clinical studies supports their potential as innovative therapeutic strategies for the prevention and management of gynecological and pregnancy-related disorders [16]. Evidence from preclinical studies, together with findings from pilot and observational clinical studies, suggests that direct microbial interventions improve gamete quality, maintain reproductive tract integrity, promote embryo development and implantation, and support gestational success [131,132,133]. Probiotic-based interventions need to consider the form of delivery to avoid undesired dissemination due to ascending colonization, hematogenous dissemination, or lymphatic transport [35]. A phase 2 randomized placebo-controlled trial demonstrated that vaginal administration of *Lactobacillus* can restore vaginal eubiosis after infection and reduce genital tract inflammation, supporting its potential as an adjunct to conventional antimicrobial therapy [134]. However, as highlighted in recent reviews, these effects appear to be strain-dependent and require confirmation in larger randomized clinical trials before routine clinical application [35,56]. Available clinical studies, including randomized controlled trials regarding vaginal probiotics administration, have generally employed doses comparable to those used for oral probiotics protocols (~10^7^ to 10^10^ colony-forming units/day), although the treatment duration is highly variable [135,136]. Vaginal probiotic strains most frequently evaluated in these clinical protocols include specific strains from species such as *L*. *reuteri*, *L. fermentum*, *L. gasseri*, *L. rhamnosus*, *L. acidophilus*, *L. crispatus*, *L. casei*, *Ligilactobacillus salivarius*, and other noncanonical vaginal *Lactobacillus* [17,35,134].

The association between vaginal microbiota dysbiosis and adverse reproductive outcomes, as well as its relevance for interpreting upper reproductive tract microbial profiles, has prompted pilot interventional studies to investigate whether probiotic therapies can restore endometrial microbial homeostasis and improve reproductive outcomes in selected patient populations. However, currently available clinical protocols remain highly heterogeneous regarding patient selection, probiotic strains, routes of administration, dosage, and treatment duration. Moreover, clinical findings are still inconsistent, highlighting the need for further optimization, standardization, and individualized patient evaluation [56].

As summarized in a recent systematic review and meta-analysis, the majority of clinical trials on the modulation of reproductive dysbiosis are carried out using oral probiotics [35]. Indeed, evidence from pilot studies and randomized clinical trials suggests that the concomitant oral administration of *Bifidobacterium* and *Lactobacillus* strains alongside conventional therapies such as metformin, oral contraceptives, or progestin-based hormonal treatments has been associated with significant improvements in the clinical manifestations of PMOS and endometriosis [35,130,137,138]. In addition, accumulating evidence may reflect that oral probiotics also influence placental development and function, as they can promote the release of beneficial metabolites and potentially translocate from the gut to the placenta [16]. During pregnancy, clinical trials evaluating probiotic supplementation, particularly formulations containing *Lactobacillus* and *Bifidobacterium* species, have primarily assessed maternal metabolic parameters, gut microbiota composition, and neonatal outcomes [108,139,140,141,142]. Although some studies have reported potential benefits for pregnancy-related complications, including preeclampsia, the available evidence remains limited and inconsistent. Therefore, it is still necessary to perform adequately powered randomized clinical trials with preeclampsia as the primary endpoint, together with additional mechanistic studies to establish the efficacy, safety, and translational potential of probiotics in gynecological and gestational medicine.

The concept of synthetic bacterial consortia has recently emerged as a novel microbiota-based therapeutic approach [143,144,145]. These consortia consist of artificially designed microbial communities composed of defined bacterial proportions with specific and complementary functional properties that collectively promote host health. In a preclinical mouse model of bacterial vaginosis, Li and colleagues developed a consortium of selected bacterial strains that significantly reduced *Gardnerella* abundance, decreased local inflammation, restored the dominance of beneficial bacteria, and improved the vaginal microenvironment. Moreover, the authors proposed that synthetic bacterial consortia may represent a safer, more controllable, and more reproducible strategy than vaginal microbiota transplantation [144]. While these preclinical findings are encouraging, whether synthetic bacterial consortia can be successfully translated into clinical practice remains an open question.

In recent years, the concept of postbiotics has gained attention as a novel microbiome-targeted therapeutic approach. In 2021, an expert panel convened by ISAPP defined postbiotics as preparations of inanimate microorganisms and/or their components that confer health benefits to the host. Although postbiotics are unable to colonize the host, they retain the capacity to modulate microbiota composition and metabolic activity [146]. Several preclinical studies have demonstrated that administration of standardized inactivated microbial strains can exert beneficial physiological and immunological effects [147,148]. Importantly, the use of non-viable microorganisms may display enhanced biosafety for vulnerable populations such as immunocompromised individuals, children, pregnant women, and breastfeeding mothers. However, given its recent emergence, robust clinical evidence in humans remains limited [146,149].

Finally, fecal and vaginal microbiota transplantation has emerged as a promising strategy for microbiota remodeling. These procedures involve the transfer of fecal or vaginal microorganisms from healthy donors to restore immune and microbial homeostasis [127,150,151]. Fecal microbiota transplantation is currently employed only in the management of *Clostridioides difficile* infection in clinical settings; other applications are a matter of research [150]. Regarding this strategy in reproductive medicine, its application remains largely unexplored. Preclinical studies have shown that fecal microbiota transplantation may improve hormonal metabolism in PMOS [152,153] and attenuate endometriotic lesion development [154]. Nonetheless, additional mechanistic and clinical investigations are necessary before this approach can be safely and effectively translated into therapeutic interventions for women with gynecological or gestational disorders. As for vaginal microbiota transplantation, Lev-Sagie and coworkers demonstrated long-term remission and restoration of *Lactobacillus*-dominated microbiota in women with recurrent and antibiotic-refractory bacterial vaginosis [151]. A subsequent study has further supported vaginal microbiota transplantation as a promising microbiota-directed therapy for recurrent vaginal dysbiosis and probably for adverse reproductive outcomes [155]. Despite its promising outcomes, current evidence remains limited to case reports, small observational studies, and preclinical models, underscoring the need for standardized protocols, long-term safety evaluation, and randomized clinical trials before routine implementation.

The following section turns to emerging and future strategies for modulating the reproductive microbiome together with the conceptual advances and translational challenges that will shape their path from molecular insight to effective clinical practice.

## 7. Novel Insights and Future Perspectives

**Figure 2:**
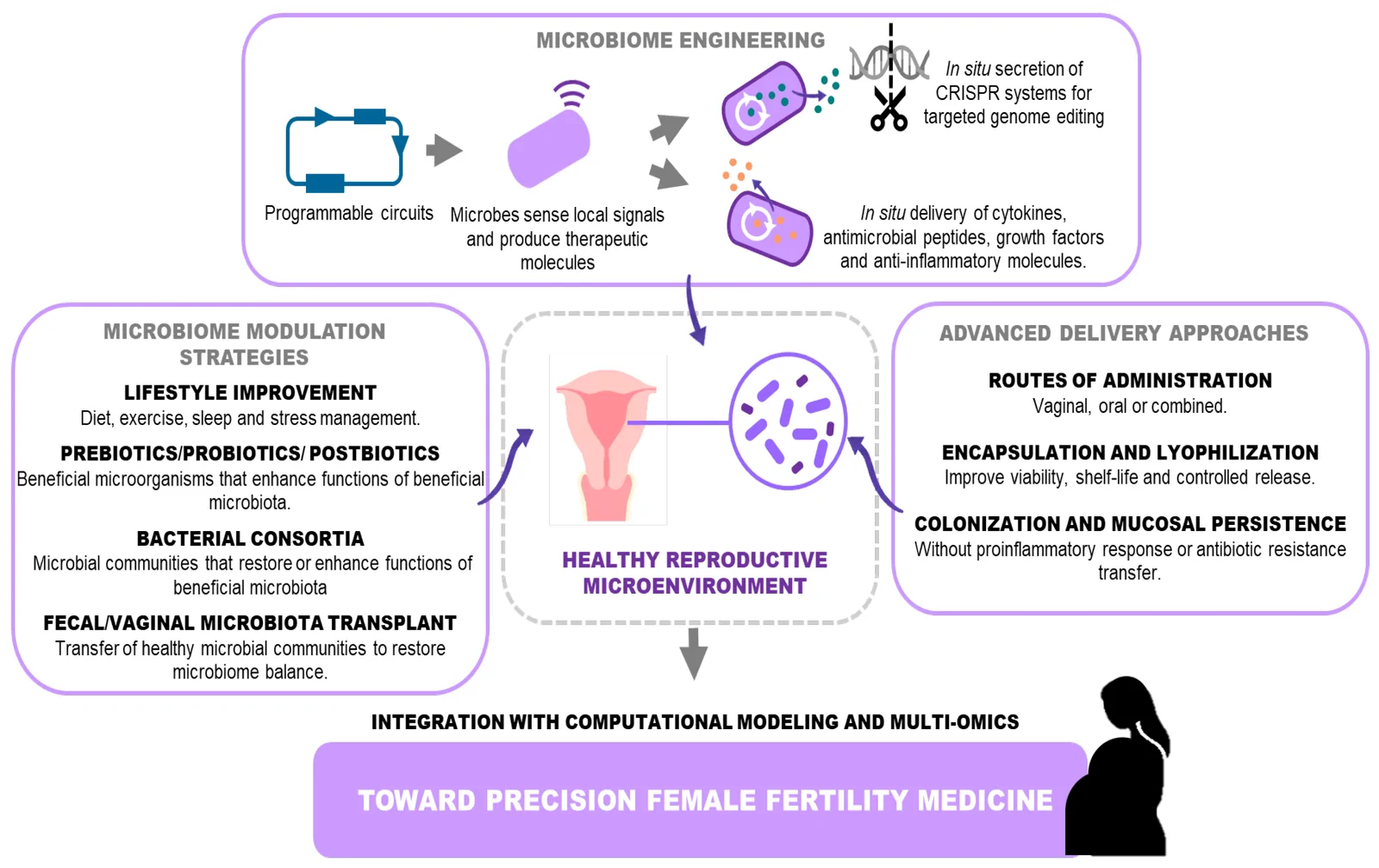
Future perspectives in microbiota modulation for female fertility. The illustration of the uterus was adapted from a CC0 image available on Wikimedia Commons.

Advances in synthetic biology and microbiome engineering are opening new avenues for the potential development of precision therapeutics targeting reproductive health. Figure 2 summarizes the microbiota-directed and bioengineering strategies discussed here and in the preceding section, spanning microbiome modulation, engineering, advanced delivery, and integration with computational and multi-omics approaches.

Beyond the conventional microbiota modulation strategies previously described, emerging approaches aim to design bioengineered microbial platforms capable of sensing, responding to, and actively regulating the reproductive microenvironment [11,12,17]. These next-generation living biotherapeutics are being explored as potential tools to restore microbial eubiosis, modulate local immune responses, and improve endometrial receptivity and maternal–fetal immunotolerance. Because *Lactobacillus* species are the principal taxa maintaining reproductive tract homeostasis, they are increasingly recognized as an auspicious reservoir of novel diagnostic and therapeutic strategies [11,17].

One of the most promising strategies is the development of engineered probiotics with enhanced colonization capacity and targeted functional properties, enabled by advances in synthetic biology such as cell-to-cell communication systems, programmable gene-regulatory platforms, and CRISPR-based control technologies [12]. Successful implementation of live biotherapeutics largely depends on maintaining microbial viability and stability during storage and delivery. Strategies such as optimized lyophilization and microencapsulation have significantly improved bacterial survival, shelf-life, and controlled mucosal release. In particular, encapsulation systems based on alginate matrices, double coatings, electrospun fibers, or mucoadhesive polymers may enhance microbial persistence within the vaginal environment while reducing administration frequency [17,156]. Moreover, the route of administration can influence therapeutic efficacy. Intravaginal delivery enables direct mucosal colonization, whereas oral administration offers greater patient compliance and may also modulate the gut–vagina/endometrial immune axis. Consequently, combined or sequential administration strategies integrating both approaches are emerging as promising alternatives [17].

Genetic editing and synthetic circuit design allow microorganisms to be programmed to produce anti-inflammatory cytokines, adhesion molecules, immunomodulatory metabolites, or antimicrobial peptides directly within the vaginal, endometrial, or intestinal environments. Although bioengineered microbiome therapeutics are still emerging, several technologies have already reached early clinical evaluation, providing preliminary evidence of their safety and feasibility. One of the examples is the engineering of *L. jensenii* to secrete the antiviral protein cyanovirin-N as a live vaginal microbicide. This strain achieved stable vaginal epithelial cell colonization without inducing mucosal inflammation, demonstrating the feasibility of genetically engineered vaginal bacteria in humans [157]. The recent initiation of the phase I trial evaluating vaginal administration of this genetically modified microorganism (MucoCept-CVN) represents an important milestone toward the clinical translation of engineered vaginal live biotherapeutics [158]. Likewise, engineered *Lactococcus lactis* secreting interleukin-10 (IL-10) successfully reduced intestinal inflammation in experimental colitis and subsequently reached Phase I clinical evaluation, establishing the safety of bacterial cytokine delivery [159,160].

Beyond these early clinical examples, a growing body of preclinical evidence supports the potential of engineered microorganisms as therapeutic platforms. In the last decade, there has been rapid growth in the use of engineered bacteria as targeted delivery vectors, particularly for tumors, providing proof of concept that commensal and probiotic strains can be programmed as therapeutic platforms. For example, *E. coli* Nissle 1917 has been engineered both to deliver anti-tumor genes to hypoxic tumor regions and to release checkpoint-blockade nanobodies locally [161,162], whereas *Lactococcus lactis* strain expressing IL-12 enhanced antitumoral responses in cervical cancer [163]. Emerging evidence also supports the application of engineered microorganisms to female reproductive health. Engineered *E. coli* Nissle 1917 alleviates intestinal injury and reproductive toxicity in rats, illustrating how microbial engineering may simultaneously target gut dysbiosis and reproductive dysfunction [164]. Likewise, engineered *L. crispatus* strains promoted endometrial regeneration and enhanced the expression of factors associated with improved fertility outcomes [165]. Genetically engineered *Lactobacillus* strains expressing anti-HIV molecules have also been developed for vaginal administration, providing proof of concept that engineered vaginal commensals can serve as safe and effective platforms for the localized delivery of therapeutic proteins [166,167]. Likewise, engineered phage-derived endolysins have emerged as a targeted antimicrobial strategy, exemplified by PM-477, which selectively lysed *Gardnerella* spp. and disrupted *Gardnerella*-dominated biofilms in ex vivo vaginal samples while preserving the protective vaginal microbiota [168]. A complementary strategy is the use of bacteria engineered to eliminate pathogens directly rather than to secrete therapeutic factors. CRISPR-Cas systems can be programmed as sequence-specific antimicrobials that remove individual strains from a mixed community while sparing commensals: phagemid-delivered CRISPR-Cas9 has been shown to selectively kill targeted bacterial pathogens in vitro [169], and conjugation-delivered CRISPR-Cas9 eliminated over 99.9% of a targeted antibiotic-resistant strain in the gut microbiota of mice in a single dose [170]. Although these approaches have not yet been applied to reproductive disorders, they provide evidence for highly selective microbiome editing.

Building on such precedents, genetically engineered *Lactobacillus* strains or other commensal microorganisms could be designed to detect molecular signatures associated with dysbiosis, pathogenic microorganisms, or inflammation-related markers and, in response, release beneficial metabolites, immunomodulatory mediators, and/or key implantation-associated factors such as IL-10, IL-22, leukemia inhibitory factor (LIF), or transforming growth factor-β (TGF-β) [17]. Likewise, CRISPR-based antimicrobial systems could potentially be adapted to selectively eliminate dysbiosis-associated taxa, including *Gardnerella* spp. and other anaerobes, thereby avoiding the collateral disruption produced by broad-spectrum antibiotics [171]. Since oral antibiotic therapy remains the standard treatment for reproductive tract infections despite frequent recurrence and co-infections [172,173], bioengineering approaches may offer alternatives that enable sustained and site-specific therapeutic release, targeted modulation of mucosal immune responses, reduced systemic exposure, and potentially improved reproductive outcomes.

Realizing these engineered strategies clinically will depend on coupling them to patient-specific guidance. Advances in computational modeling and multi-omics integration are increasingly able to identify patient-specific microbial and immune signatures that could determine which intervention suits which patient, transforming generic microbiota modulation into personalized, mechanistically targeted therapy [174].

In parallel with microbiota engineering, advances in reproductive tissue technology are enabling the development of biomimetic platforms capable of reproducing key structural, hormonal, and immunological features of the female reproductive tract. Engineered reproductive tissues, organoids, and microfluidic ‘organ-on-chip’ systems are emerging as promising tools for studying implantation, endometrial receptivity, embryo–maternal communication, and microbiota–host interactions under controlled conditions. These systems also hold future potential for personalized reproductive medicine and therapeutic development [175,176,177]. Therapeutic approaches discussed in this review are classified by their current stage of development and summarized in Table 3.

Overall, while selected engineered live biotherapeutics have already entered early-phase clinical testing [158,160], most microbiome engineering strategies for reproductive disorders remain at the proof-of-concept or preclinical stage. Collectively, these approaches have the potential to drive a paradigm shift toward precision fertility medicine, in which bioengineered microbiota therapeutics and advanced tissue-engineering platforms could eventually enable the prevention, study, and treatment of infertility, implantation failure, recurrent pregnancy loss, and gestational complications.

Although engineered microorganisms, synthetic microbial consortia, and other living biotherapeutics represent promising platforms for precision medicine, their translation into reproductive medicine remains highly challenging. Unlike other therapeutic contexts, interventions targeting the female reproductive tract involve unique safety considerations. Unresolved issues include the long-term ecological stability of engineered microorganisms, the possibility of horizontal transfer of genetic elements, unwanted interactions with resident microbial communities, host immune responses, and regulatory challenges associated with the administration of genetically modified organisms during the reproductive period [178]. The clinical translation of engineered live biotherapeutics requires rigorous demonstration of product quality, including the safety, reliability, robustness, and batch-to-batch consistency of the manufacturing process. Safety assessment is particularly critical because bacterial chassis may contain immunostimulatory components, whose biological effects can vary depending on the route of administration and tissue exposure. Reproductive medicine imposes stringent safety requirements [178]. Any potential dissemination of engineered microorganisms or their bioactive products beyond the intended site must be carefully excluded, as unintended exposure could interfere with embryo implantation, placental development, maternal–fetal immune tolerance, or fetal health. Likewise, horizontal transfer of engineered genetic elements and uncontrolled microbial persistence or proliferation within the reproductive tract represent additional biosafety concerns that must be minimized through robust genetic safeguards and biocontainment strategies designed to restrict microbial survival and replication. Consequently, most bioengineered microbiome-based strategies should currently be considered experimental, requiring extensive preclinical validation, carefully designed clinical studies, and stringent regulatory evaluation before potential application in infertility or pregnancy-related disorders.

## 8. Discussion

Growing evidence indicates that the female reproductive tract microbiota is not merely a passive microbial niche but a dynamic and functionally active ecosystem that may contribute to fertility, implantation, pregnancy maintenance, and maternal–fetal health. The integration of microbiome research with immunology, metabolism, endocrinology, and reproductive biology is providing new perspectives on the pathophysiology of infertility and reproductive disorders. At the same time, these findings support the hypothesis of complex interactions between microbial communities and reproductive physiology, increasingly an emerging gut–vagina–endometrium axis that links distant mucosal sites through immune, metabolic, and endocrine pathways.

Despite remarkable technological progress, the field remains limited by substantial methodological heterogeneity, low-biomass sampling challenges, incomplete causal evidence, and the lack of standardized clinical frameworks. These constraints bear directly on the use of the microbiome as a diagnostic tool: although microbial signatures show clear promise as predictive and prognostic biomarkers of reproductive outcomes, their clinical translation has been limited by inconsistent findings across cohorts and the absence of harmonized criteria. A recurring lesson from these studies is that taxonomic composition alone is insufficient, and that functional and ecological characterization, for example distinguishing the distinct reproductive roles of different *Lactobacillus* species, may ultimately carry greater predictive value. Future advances will require large, multicenter longitudinal studies, integrated analytical protocols, contamination-aware methodologies, and clinically validated biomarkers capable of distinguishing physiological microbial variation from true dysbiosis. Reproductive microbiome research must evolve toward integrative models that simultaneously consider the vaginal, endometrial, and gut microbiomes, as well as the host’s immune, metabolic, hormonal, and environmental factors. Furthermore, although this review focuses exclusively on the female reproductive microbiome, accumulating evidence suggests that the male reproductive microbiome may be associated with fertility-related parameters, including sperm physiology [179], capacitation, motility, DNA integrity, and interactions with the female reproductive tract after insemination. Elucidating the bidirectional contribution of both partners’ microbiomes to reproductive success represents an important direction for future research.

From a translational perspective, microbiota-directed interventions, including probiotics, prebiotics, postbiotics, synthetic microbial consortia, gut and vaginal microbiota transplantation, and engineered living biotherapeutics, represent promising but still largely experimental strategies (Figure 2). Although the microbiota represents an attractive therapeutic target, current evidence does not yet support the routine clinical use of microbiome testing or probiotic interventions to improve ART outcomes. While selected probiotic interventions and a limited number of engineered living biotherapeutics have reached early clinical evaluation, most microbiome-based reproductive therapies remain at the preclinical or proof-of-concept stage, and robust evidence supporting their efficacy in reproductive medicine is still limited. Advances in synthetic biology, CRISPR-based engineering, systems biology, and reproductive tissue bioengineering provide a conceptual framework that could eventually enable the development of programmable microbial therapeutics capable of locally delivering immunomodulatory molecules, metabolites, or implantation-supporting factors. Before these technologies can be incorporated into routine clinical practice, additional mechanistic studies together with adequately powered randomized clinical trials will be required to establish their efficacy, safety, and long-term reproductive outcomes. Furthermore, because reproductive medicine demands exceptionally high safety standards, future clinical translation will require rigorous validation of ecological stability, genomic safety, and long-term reproductive outcomes.

## 9. Conclusions

The female reproductive microbiome represents a promising but still evolving field of research with potential implications for reproductive diagnostics and therapeutics. While increasing research provides preliminary evidence supporting important interactions between microbial communities and reproductive physiology, substantial methodological heterogeneity, unresolved mechanistic questions, and limited clinical validation currently restrict routine clinical implementation of microbiome-based diagnostic tests or therapeutic interventions to improve reproductive outcomes. Future progress will depend on standardized analytical approaches, multicenter validation studies, and adequately powered randomized placebo-controlled clinical trials to establish the efficacy, safety, and clinical utility of microbiome-based interventions. In parallel, continued advances in immunology, systems biology, multi-omics integration, and bioengineering, particularly the application of synthetic biology to engineer beneficial, health-promoting microbiomes, may contribute to the future development of more precise, individualized, and evidence-based strategies to improve female reproductive healthcare. Nevertheless, realizing this potential will require additional mechanistic insights, along with adequately powered clinical trials, before these approaches can be safely and effectively incorporated into routine clinical practice.

## Figures and Tables

**Table 3:** Current translational status of microbiome-based therapeutic strategies for female reproductive health.

Therapeutic Strategy	Current Stage of Development	Representative Evidence/Examples	Current Status in Reproductive Medicine
Probiotics	Early Clinical Evaluation * [135,136]	*Lactobacillus*-based formulations evaluated in infertility, recurrent bacterial vaginosis, ART, and pregnancy-related disorders.	Used clinically for selected indications *, although evidence supporting routine use to improve fertility or ART outcomes remains limited.
Postbiotics	Proof-of-Concept//Preclinical Studies [124,125]	Experimental studies evaluating microbial metabolites and inactivated bacterial products.	Insufficient evidence for reproductive applications.
Vaginal Microbiota Transplantation (VMT)	Case Reports, Observational Studies/Preclinical models [144,151,155]	First human studies for recurrent bacterial vaginosis	Not standard of care in reproductive medicine yet.
Fecal Microbiota Transplantation (FMT)	Clinical Practice [150,152,153,154]	Established therapy for recurrent *Clostridioides difficile* infection; reproductive applications remain experimental (preclinical)	No established indication in reproductive medicine yet.
Engineered Live Biotherapeutics (engineered *Lactobacillus*, *L. jensenii*, *L. lactis*)	Early clinical evaluation * [158,159,160]	MucoCept-CVN (Phase I); IL-10-secreting *Lactococcus lactis* (Phase I)	Early clinical translation demonstrating feasibility and safety; no reproductive indication approved *.
Engineered Live Biotherapeutics (CRISPR, engineered commensals/probiotics, synthetic microbial consortia)	Proof-of-Concept/ Preclinical studies [164,165,170]	Engineered *E. coli* Nissle 1917, engineered *L. crispatus*, and CRISPR-Cas antimicrobial systems	Preclinical studies only.
Engineered phage-derived endolysins	Proof-of-Concept [168]	PM-477 targeting *Gardnerella* biofilms	Experimental strategy with encouraging ex vivo results.
Reproductive tissue technology (organoids, organ-on-chip systems)	Proof-of-Concept [175,176,177]	Endometrial organoids and reproductive microphysiological systems	Experimental platforms for mechanistic studies and future personalized medicine.

* Routine microbiome-based treatment to improve fertility or assisted reproductive technology outcomes is not currently supported by sufficient evidence.

## Data Availability

No new data were created or analyzed in this study. Data sharing is not applicable to this article.

## References

[1] Bhattacharjee N., Schumacher A., Aali A., Habtegiorgis Abate Y., Abbasgholizadeh R., Abbasian M., Abbasi-Kangevari M., Abbastabar H., ElHafeez S., Abd-Elsalam A. (2024). Global fertility in 204 countries and territories, 1950–2021, with forecasts to 2100: A comprehensive demographic analysis for the Global Burden of Disease Study 2021. Lancet.

[2] World Health Organization Infertility Prevalence Estimates, 1990–2021. https://www.who.int/publications/i/item/978920068315.

[3] Wyns C., De Geyter C., Calhaz-Jorge C., Kupka M.S., Motrenko T., Smeenk J., Bergh C., Tandler-Schneider A., European IVF Monitoring Consortium (EIM), European Society of Human Reproduction and Embryology (ESHRE) (2022). ART in Europe, 2018: Results generated from European registries by ESHRE. Hum. Reprod. Open.

[4] Wang N., Chen L., Yi K., Zhang B., Li C., Zhou X. (2024). The effects of microbiota on reproductive health: A review. Crit. Rev. Food Sci. Nutr..

[5] Huang T., Liang X., Bao H., Ma G., Tang X., Luo H., Xiao X. (2024). Multi-omics analysis reveals the associations between altered gut microbiota, metabolites, and cytokines during pregnancy. mSystems.

[6] Sun Y., Gao S., Ye C., Zhao W. (2023). Gut microbiota dysbiosis in polycystic ovary syndrome: Mechanisms of progression and clinical applications. Front. Cell. Infect. Microbiol..

[7] Salliss M.E., Farland L.V., Mahnert N.D., Herbst-Kralovetz M.M. (2021). The role of gut and genital microbiota and the estrobolome in endometriosis, infertility and chronic pelvic pain. Hum. Reprod. Update.

[8] Ridder A., Giorgione V., Khalil A., Thilaganathan B. (2019). Preeclampsia: The Relationship between Uterine Artery Blood Flow and Trophoblast Function. Int. J. Mol. Sci..

[9] Pollheimer J., Vondra S., Baltayeva J., Beristain A.G., Knöfler M. (2018). Regulation of Placental Extravillous Trophoblasts by the Maternal Uterine Environment. Front. Immunol..

[10] Lopez-Tello J., Schofield Z., Kiu R., Dalby M.J., van Sinderen D., Le Gall G., Sferruzzi-Perri A.N., Hall L.J. (2022). Maternal gut microbiota Bifidobacterium promotes placental morphogenesis, nutrient transport and fetal growth in mice. Cell Mol. Life Sci..

[11] Narendrakumar L., Dhiman P., Das B. (2026). The human reproductive tract microbiome: A novel source of live biotherapeutics. Prog. Mol. Biol. Transl. Sci..

[12] Bober J.R., Beisel C.L., Nair N.U. (2018). Synthetic Biology Approaches to Engineer Probiotics and Members of the Human Microbiota for Biomedical Applications. Annu. Rev. Biomed. Eng..

[13] Miyagi M., Mekaru K., Tanaka S.E., Arai W., Ashikawa K., Sakuraba Y., Nakamura R., Oishi S., Akamine K., Aoki Y. (2023). Endometrial and vaginal microbiomes influence assisted reproductive technology outcomes. JBRA Assist Reprod..

[14] Moreno I., Garcia-Grau I., Perez-Villaroya D., Gonzalez-Monfort M., Bahçeci M., Barrionuevo M.J., Taguchi S., Puente E., Dimattina M., Lim M.W. (2022). Endometrial microbiota composition is associated with reproductive outcome in infertile patients. Microbiome.

[15] Wang M., Zheng L.W., Ma S., Zhao D.H., Xu Y. (2024). The gut microbiota: Emerging biomarkers and potential treatments for infertility-related diseases. Front. Cell. Infect. Microbiol..

[16] Feng T., Liu Y. (2022). Microorganisms in the reproductive system and probiotic’s regulatory effects on reproductive health. Comput. Struct. Biotechnol. J..

[17] Li L., Feng L., Li Q., Zhou Y., Liu S., Song H., Kang S., Chen H., Cong H., Liu S. (2026). Precision Microbial Therapeutics for Infertility: Next-Generation Probiotics, Engineered Biologics and Translational Pathways. Microb. Biotechnol..

[18] Tuniyazi M., Zhang N. (2023). Possible Therapeutic Mechanisms and Future Perspectives of Vaginal Microbiota Transplantation. Microorganisms.

[19] Salminen S., Collado M.C., Endo A., Hill C., Lebeer S., Quigley E.M.M., Sanders M.E., Shamir R., Swann J.R., Szajewska H. (2021). The International Scientific Association of Probiotics and Prebiotics (ISAPP) consensus statement on the definition and scope of postbiotics. Nat. Rev. Gastroenterol. Hepatol..

[20] Zhang H., Zha X., Zhang B., Zheng Y., Elsabagh M., Wang H. (2024). Gut microbiota contributes to bisphenol A-induced maternal intestinal and placental apoptosis, oxidative stress, and fetal growth restriction in pregnant ewe model by regulating gut-placental axis. Microbiome.

[21] Jin J., Gao L., Zou X., Zhang Y., Zheng Z., Zhang X. (2022). Gut Dysbiosis Promotes Preeclampsia by Regulating Macrophages and Trophoblasts. Circ. Res..

[22] Aggarwal N., Kitano S., Puah G.R.Y., Kittelmann S., Hwang I.Y., Chang M.W. (2023). Microbiome and Human Health: Current Understanding, Engineering, and Enabling Technologies. Chem. Rev..

[23] Cho I., Blaser M. (2012). The human microbiome: At the interface of health and disease. Nat. Rev. Genet..

[24] Elkafas H., Walls M., Al-Hendy A., Ismail N. (2023). Gut and genital tract microbiomes: Dysbiosis and link to gynecological disorders. Front. Cell. Infect. Microbiol..

[25] Moreno I., Codoñer F.M., Vilella F., Valbuena D., Martinez-Blanch J.F., Jimenez-Almazán J., Alonso R., Alamá P., Remohí J., Pellicer A. (2016). Evidence that the endometrial microbiota has an effect on implantation success or failure. Am. J. Obstet. Gynecol..

[26] Condori-Catachura S., Ahannach S., Ticlla M., Kenfack J., Livo E., Anukam K.C., Pinedo-Cancino V., Collado M.C., Dominguez-Bello M.G., Miller C. (2025). Diversity in women and their vaginal microbiota. Trends Microbiol..

[27] Odendaal J., Black N., Bennett P.R., Brosens J., Quenby S., MacIntyre D.A. (2024). The endometrial microbiota and early pregnancy loss. Hum. Reprod..

[28] Zhang Z., Zhang Y., Wu A., Xu C., Li Z. (2025). Will gut, oral, and vaginal microbiota influence the outcome of FET or be influenced by FET? A pilot study. mBio.

[29] Chen C., Song X., Wei W., Zhong H., Dai J., Lan Z., Li F., Yu X., Feng Q., Wang Z. (2017). The microbiota continuum along the female reproductive tract and its relation to uterine-related diseases. Nat. Commun..

[30] Mitchell C.M., Haick A., Nkwopara E., Garcia R., Rendi M., Agnew K., Fredricks D.N., Eschenbach D. (2015). Colonization of the upper genital tract by vaginal bacterial species in nonpregnant women. Am. J. Obstet. Gynecol..

[31] Tao X., Franasiak J., Zhan Y., Scott R., Rajchel J., Bedard J., Newby R., Scott R., Treff R., Chu T. (2017). Characterizing the endometrial microbiome by analyzing the ultra-low bacteria from embryo transfer catheter tips in IVF cycles: Next generation sequencing (NGS) analysis of the 16S ribosomal gene. Hum. Microb. J..

[32] Sola-Leyva A., Andrés-León E., Molina N.M., Terron-Camero L.C., Plaza-Díaz J., Sáez-Lara M.J., Gonzalvo M.C., Sánchez R., Ruíz S., Martínez L. (2021). Mapping the entire functionally active endometrial microbiota. Hum. Reprod..

[33] Xie Z., Chen Z., Chai Y., Yao W., Ma G. (2025). Unveiling the placental bacterial microbiota: Implications for maternal and infant health. Front. Physiol..

[34] Cariati F., Carotenuto C., Bagnulo F., Pacella D., Marrone V., Paolillo R., Catania M.R., Di Girolamo R., Conforti A., Strina I. (2024). Endometrial microbiota profile in in-vitro fertilization (IVF) patients by culturomics-based analysis. Front. Endocrinol..

[35] López-Moreno A., Aguilera M. (2021). Vaginal Probiotics for Reproductive Health and Related Dysbiosis: Systematic Review and Meta-Analysis. J. Clin. Med..

[36] de Goffau M.C., Lager S., Sovio U., Gaccioli F., Cook E., Peacock S.J., Parkhill J., Charnock-Jones D.S., Smith G.C.S. (2019). Human placenta has no microbiome but can contain potential pathogens. Nature.

[37] Panzer J.J., Romero R., Greenberg J.M., Winters A.D., Galaz J., Gomez-Lopez N., Theis K.R. (2023). Is there a placental microbiota? A critical review and re-analysis of published placental microbiota datasets. BMC Microbiol..

[38] Payne M.S., Bayatibojakhi S. (2014). Exploring preterm birth as a polymicrobial disease: An overview of the uterine microbiome. Front. Immunol..

[39] Satokari R., Grönroos T., Laitinen K., Salminen S., Isolauri E. (2009). Bifidobacterium and Lactobacillus DNA in the human placenta. Lett. Appl. Microbiol..

[40] Hu J., Benny P., Wang M., Ma Y., Lambertini L., Peter I., Xu Y., Lee M.J. (2021). Intrauterine Growth Restriction Is Associated with Unique Features of the Reproductive Microbiome. Reprod. Sci..

[41] Bhattacharya K., Dutta S., Sengupta P., Bagchi S. (2023). Reproductive tract microbiome and therapeutics of infertility. Middle East Fertil. Soc. J..

[42] Rautava S., Luoto R., Salminen S., Isolauri E. (2012). Microbial contact during pregnancy, intestinal colonization and human disease. Nat. Rev. Gastroenterol. Hepatol..

[43] Aagaard K., Ma J., Antony K.M., Ganu R., Petrosino J., Versalovic J. (2014). The placenta harbors a unique microbiome. Sci. Transl. Med..

[44] Inversetti A., Zambella E., Guarano A., Dell’Avanzo M., Di Simone N. (2023). Endometrial Microbiota and Immune Tolerance in Pregnancy. Int. J. Mol. Sci..

[45] Santos Aleman R., Moncada M., Aryana K.J. (2023). Leaky Gut and the Ingredients That Help Treat It: A Review. Molecules.

[46] Ziętek M., Celewicz Z., Szczuko M. (2021). Short-Chain Fatty Acids, Maternal Microbiota and Metabolism in Pregnancy. Nutrients.

[47] Mann E.R., Lam Y.K., Uhlig H.H. (2024). Short-chain fatty acids: Linking diet, the microbiome and immunity. Nat. Rev. Immunol..

[48] Tomasova L., Konopelski P., Ufnal M. (2016). Gut Bacteria and Hydrogen Sulfide: The New Old Players in Circulatory System Homeostasis. Molecules.

[49] Wallace K.L., Zheng L.B., Kanazawa Y., Shih D.Q. (2014). Immunopathology of inflammatory bowel disease. World J. Gastroenterol..

[50] Amabebe E., Anumba D.O. (2022). Diabetogenically beneficial gut microbiota alterations in third trimester of pregnancy. Reprod. Fertil..

[51] Rezasoltani S., Yadegar A., Asadzadeh Aghdaei H., Reza Zali M. (2021). Modulatory effects of gut microbiome in cancer immunotherapy: A novel paradigm for blockade of immune checkpoint inhibitors. Cancer Med..

[52] Qiu Q., Lin Y., Ma Y., Li X., Liang J., Chen Z. (2021). Exploring the Emerging Role of the Gut Microbiota and Tumor Microenvironment in Cancer Immunotherapy. Front. Immunol..

[53] Chakraborty P., Gamage H.K.A.H., Laird A.S. (2024). Butyrate as a potential therapeutic agent for neurodegenerative disorders. Neurochem. Int..

[54] Zundler S., Günther C., Kremer A.E., Zaiss M.M., Rothhammer V., Neurath M.F. (2023). Gut immune cell trafficking: Inter-organ communication and immune-mediated inflammation. Nat. Rev. Gastroenterol. Hepatol..

[55] Teede H.J., Khomami M.B., Morman R., Laven J.S.E., Joham A.E., Costello M.F., Patil M., Rees D.A., Berry L., Cree M.G. (2026). Global Name Change Consortium. Polyendocrine metabolic ovarian syndrome, the new name for polycystic ovary syndrome: A multistep global consensus process. Lancet.

[56] Hiratsuka D., Matsuo M., Hirota Y. (2026). The reproductive tract microbiome and female fertility: Dysbiosis, disease links, and emerging therapeutic strategies. Fertil. Steril..

[57] Zambella E., Peruffo B., Guarano A., Inversetti A., Di Simone N. (2024). The Hidden Relationship between Intestinal Microbiota and Immunological Modifications in Preeclampsia Pathogenesis. Int. J. Mol. Sci..

[58] Hu P., Chen X., Chu X., Fan M., Ye Y., Wang Y. (2021). Association of Gut Microbiota during Early Pregnancy with Risk of Incident Gestational Diabetes Mellitus. J. Clin. Endocrinol. Metab..

[59] Koedooder R., Singer M., Schoenmakers S., Savelkoul P.H.M., Morré S.A., de Jonge J.D., Poort L., Cuypers W.J.S.S., Beckers N.G.M., Broekmans F.J.M. (2019). The vaginal microbiome as a predictor for outcome of in vitro fertilization with or without intracytoplasmic sperm injection: A prospective study. Hum. Reprod..

[60] Vomstein K., Krog M.C., Wrønding T., Nielsen H.S. (2024). The microbiome in recurrent pregnancy loss—A scoping review. J. Reprod. Immunol..

[61] Barrientos G., Ronchi F., Conrad M.L. (2024). Nutrition during pregnancy: Influence on the gut microbiome and fetal development. Am. J. Reprod. Immunol..

[62] Gao X., Louwers Y.V., Laven J.S.E., Schoenmakers S. (2024). Clinical Relevance of Vaginal and Endometrial Microbiome Investigation in Women with Repeated Implantation Failure and Recurrent Pregnancy Loss. Int. J. Mol. Sci..

[63] Garmendia J.V., De Sanctis C.V., Hajdúch M., De Sanctis J.B. (2024). Microbiota and Recurrent Pregnancy Loss (RPL); More than a Simple Connection. Microorganisms.

[64] Tersigni C., D’Ippolito S., Di Nicuolo F., Marana R., Valenza V., Masciullo V. (2019). Recurrent pregnancy loss is associated to leaky gut: A novel pathogenic model of endometrium inflammation?. J. Transl. Med..

[65] Alsharairi N.A., Li L. (2023). Gut Microbiota, Inflammation, and Probiotic Supplementation in Fetal Growth Restriction—A Comprehensive Review of Human and Animal Studies. Life.

[66] Tang R., Xiao G., Jian Y., Yuan Q., Jiang C., Wang W. (2022). The Gut Microbiota Dysbiosis in Preeclampsia Contributed to Trophoblast Cell Proliferation, Invasion, and Migration via lncRNA BC030099/NF-κB Pathway. Mediat. Inflamm..

[67] Ata B., Yildiz S., Turkgeldi E., Brocal V.P., Dinleyici E.C., Moya A. (2019). The Endobiota Study: Comparison of Vaginal, Cervical and Gut Microbiota Between Women with Stage 3/4 Endometriosis and Healthy Controls. Sci. Rep..

[68] Hernandes C., Silveira P., Rodrigues Sereia A.F., Christoff A.P., Mendes H., Valter de Oliveira L.F., Podgaec S. (2020). Microbiome Profile of Deep Endometriosis Patients: Comparison of Vaginal Fluid, Endometrium and Lesion. Diagnostics.

[69] Svensson A., Brunkwall L., Roth B., Orho-Melander M., Ohlsson B. (2021). Associations Between Endometriosis and Gut Microbiota. Reprod. Sci..

[70] Lu C., Wang H., Yang J., Zhang X., Chen Y., Feng R., Qian Y. (2021). Changes in Vaginal Microbiome Diversity in Women With Polycystic Ovary Syndrome. Front. Cell. Infect. Microbiol..

[71] Hong X., Qin P., Huang K., Ding X., Ma J., Xuan Y., Zhu X., Peng D., Wang B. (2020). Association between polycystic ovary syndrome and the vaginal microbiome: A case-control study. Clin. Endocrinol..

[72] Gu Y., Zhou G., Zhou F., Li Y., Wu Q., He H., Zhang Y., Ma C., Ding J., Hua K. (2022). Gut and Vaginal Microbiomes in PCOS: Implications for Women’s Health. Front. Endocrinol..

[73] Tu Y., Zheng G., Ding G., Wu Y., Xi J., Ge Y., Gu H., Wang Y., Sheng J., Liu X. (2021). Comparative Analysis of Lower Genital Tract Microbiome Between PCOS and Healthy Women. Front. Physiol..

[74] Zou Y., Liao R., Cheng R., Chung H., Zhu H., Huang Y. (2023). Alterations of gut microbiota biodiversity and relative abundance in women with PCOS: A systematic review and meta-analysis. Microb. Pathog..

[75] Zhou L., Ni Z., Cheng W., Yu J., Sun S., Zhai D., Yu C., Cai Z. (2020). Characteristic gut microbiota and predicted metabolic functions in women with PCOS. Endocr. Connect..

[76] Lindheim L., Bashir M., Münzker J., Trummer C., Zachhuber V., Leber B., Horvath A., Pieber T.R., Gorkiewicz G., Stadlbauer V. (2017). Alterations in Gut Microbiome Composition and Barrier Function Are Associated with Reproductive and Metabolic Defects in Women with Polycystic Ovary Syndrome (PCOS): A Pilot Study. PLoS ONE.

[77] Wang R., Cao Q., Qiao X., Zhong Y., Bo W., Huang X., Li Y., Huang W. (2026). Vaginal and endometrial microbiota dysbiosis in patients with chronic endometritis: A systematic review and meta-analysis. Front. Cell. Infect. Microbiol..

[78] Zhang H., Zou H., Zhang C., Zhang S. (2024). Chronic endometritis and the endometrial microbiota: Implications for reproductive success in patients with recurrent implantation failure. Ann. Clin. Microbiol. Antimicrob..

[79] Xue G., Zheng Z., Liang X., Zheng Y., Wu H. (2023). Uterine Tissue Metabonomics Combined with 16S rRNA Gene Sequencing To Analyze the Changes of Gut Microbiota in Mice with Endometritis and the Intervention Effect of Tau Interferon. Microbiol. Spectr..

[80] Zhao C., Bao L., Qiu M., Feng L., Chen L., Liu Z. (2022). Dietary Tryptophan-Mediated Aryl Hydrocarbon Receptor Activation by the Gut Microbiota Alleviates Escherichia coli-Induced Endometritis in Mice. Microbiol. Spectr..

[81] Hu X., Mu R., Xu M., Yuan X., Jiang P., Guo J. (2020). Gut microbiota mediate the protective effects on endometritis induced by Staphylococcus aureus in mice. Food Funct..

[82] Lin C.Y., Lin C.Y., Yeh Y.M., Yang L.Y., Lee Y.S., Chao A., Chin C.Y., Chao A.S., Yang C.Y. (2020). Severe preeclampsia is associated with a higher relative abundance of Prevotella bivia in the vaginal microbiota. Sci. Rep..

[83] Wallace J.G., Bellissimo C.J., Yeo E., Fei Xia Y., Petrik J.J., Surette M.G. (2019). Obesity during pregnancy results in maternal intestinal inflammation, placental hypoxia, and alters fetal glucose metabolism at mid-gestation. Sci. Rep..

[84] Ahmadian E., Rahbar Saadat Y., Hosseiniyan Khatibi S.M., Nariman-Saleh-Fam Z., Bastami M., Zununi Vahed F. (2020). Pre-Eclampsia: Microbiota possibly playing a role. Pharmacol. Res..

[85] Chen X., Li P., Liu M., Zheng H., He Y., Chen M.X. (2020). Gut dysbiosis induces the development of pre-eclampsia through bacterial translocation. Gut.

[86] Colonetti T., Limas Carmo Teixeira D., Grande A.J., Rodrigues Uggioni M.L., Generoso J. (2023). The role of intestinal microbiota on pre-eclampsia: Systematic review and meta-analysis. Eur. J. Obstet. Gynecol. Reprod. Biol..

[87] Miao T., Yu Y., Sun J., Ma A., Yu J., Cui M., Yang L., Wang H. (2021). Decrease in abundance of bacteria of the genus Bifidobacterium in gut microbiota may be related to pre-eclampsia progression in women from East China. Food Nutr. Res..

[88] Lv L.J., Li S.H., Li S.C., Zhong Z.C., Duan H.L., Tian C. (2019). Early-Onset Preeclampsia Is Associated With Gut Microbial Alterations in Antepartum and Postpartum Women. Front. Cell. Infect. Microbiol..

[89] Chang Y., Chen Y., Zhou Q., Wang C., Chen L., Di W., Zhang Y. (2020). Short-chain fatty acids accompanying changes in the gut microbiome contribute to the development of hypertension in patients with preeclampsia. Clin. Sci..

[90] Altemani F., Barrett H.L., Gomez-Arango L., Josh P., David McIntyre H., Callaway L.K., Morrison M., Tyson G.W., Dekker Nitert M. (2021). Pregnant women who develop preeclampsia have lower abundance of the butyrate-producer Coprococcus in their gut microbiota. Pregnancy Hypertens..

[91] Li P., Wang H., Guo L., Gou X., Chen G., Lin D., Fan D., Guo X., Liu Z. (2022). Association between gut microbiota and preeclampsia-eclampsia: A two-sample Mendelian randomization study. BMC Med..

[92] Decout A., Krasias I., Roberts L., Gimeno Molina B., Charenton C., Brown Romero D., Tee Q.Y., Marchesi J.R., Ng S., Sykes L. (2024). Lactobacillus crispatus S-layer proteins modulate innate immune response and inflammation in the lower female reproductive tract. Nat. Commun..

[93] Lozano F.M., Lledó B., Morales R., Cascales A., Hortal M., Bernabeu A., Bernabeu R. (2023). Characterization of the Endometrial Microbiome in Patients with Recurrent Implantation Failure. Microorganisms.

[94] Gao L., Xu Q.H., Ma L.N., Luo J., Muyayalo K.P., Wang L.L., Huang D.H., Xiao X.J., Cheng S.B., Mor G. (2022). Trophoblast-derived Lactic Acid Orchestrates Decidual Macrophage Differentiation via SRC/LDHA Signaling in Early Pregnancy. Int. J. Biol. Sci..

[95] Bloom S.M., Mafunda N.A., Woolston B.M., Hayward M.R., Frempong J.F., Abai A.B., Xu J., Mitchell A.J., Westergaard X., Hussain F.A. (2022). Cysteine dependence of Lactobacillus iners is a potential therapeutic target for vaginal microbiota modulation. Nat. Microbiol..

[96] Zhou R., Lu J., Wang J., Xiao B. (2022). Vaginal Lactobacillus iners abundance is associated with outcome in antibiotic treatment of bacterial vaginosis and capable of inhibiting Gardnerella. Front. Cell. Infect. Microbiol..

[97] Chen P., Chen P., Guo Y., Fang C., Li T. (2021). Interaction Between Chronic Endometritis Caused Endometrial Microbiota Disorder and Endometrial Immune Environment Change in Recurrent Implantation Failure. Front. Immunol..

[98] Perrotta A.R., Borrelli G.M., Martins C.O., Kallas E.G., Sanabani S.S., Griffith L.G., Alm E.J., Abrao M.S. (2020). The Vaginal Microbiome as a Tool to Predict rASRM Stage of Disease in Endometriosis: A Pilot Study. Reprod. Sci..

[99] Kalı Z., Karlı P., Tanılır F., Kırıcı P., Ege S. (2025). NF-κB as an Inflammatory Biomarker in Thin Endometrium: Predictive Value for Live Birth in Recurrent Implantation Failure. Diagnostics.

[100] Fauser B.C.J.M., Adamson G.D., Boivin J., Chambers G.M., de Geyter C., Dyer S., Inhorn M.C., Schmidt L., Serour G.I., Tarlatzis B. (2024). Contributors and members of the IFFS Demographics and Access to Care Review Board. Declining global fertility rates and the implications for family planning and family building: An IFFS consensus document based on a narrative review of the literature. Hum. Reprod. Update.

[101] Samama M., Ueno J., Carvalho de Arruda Veiga E., Piscopo R.C.C.P., Ikeda F., Pires de Lemos N., Tadeu Bidinotto L., Guimarães da Silva M., Jarmy Di Bella Z., Entezami F. (2025). Vaginal Microbiome and Its Relationship with Assisted Reproduction: A Systematic Review and Meta-Analysis. Life.

[102] Foteinidou P., Exindari M., Chatzidimitriou D., Gioula G. (2024). Endometrial Microbiome and Its Correlation to Female Infertility: A Systematic Review and Meta-Analysis. Acta Microbiol. Hell..

[103] Han A.R. (2025). Endometrial microbiome in reproductive failure: The possibility of metagenomic analysis. Clin. Exp. Reprod. Med..

[104] van den Tweel M.M., van den Munckhof E.H.A., van der Zanden M., Molijn A.C., van Lith J.M.M., Le Cessie S., Boers K.E. (2024). Bacterial vaginosis in a subfertile population undergoing fertility treatments: A prospective cohort study. J. Assist. Reprod. Genet..

[105] Koort K., Sõsa K., Türk S., Lapp E., Talving E., Karits P., Rosenstein K., Jaagura M., Sekavin A., Sõritsa D. (2023). Lactobacillus crispatus-dominated vaginal microbiome and Acinetobacter-dominated seminal microbiome support beneficial ART outcome. Acta Obstet. Gynecol. Scand..

[106] Zhang J.X., Li Q.L., Wang X.Y., Zhang C.C., Chen S.T., Liu X.H., Dong X.Y., Zhao H., Huang D.H. (2024). Causal Link between Gut Microbiota and Infertility: A Two-sample Bidirectional Mendelian Randomization Study. Curr. Med. Sci..

[107] Liu X., Bin C., Gao Q., Li M., Xue L., Zhou Z., Liu M., Ji X., Wei S. (2026). Multi-omics biomarkers in female fertility: From oocyte quality to endometrial receptivity and clinical translation. Biomark. Res..

[108] Tahtouh Zaatar M., Othman R., Abushawish M., Akl M., Alachkar M.T., Almatboona G., Alriyami F., Alshaibani A., Ashkanani D., Basharova M. (2026). The Women’s Microbiome: Molecular Insights, Clinical Gaps, and Future Frontiers in Precision Health with Implications for Gulf Cooperation Council Populations. Int. J. Mol. Sci..

[109] Sobstyl A., Chałupnik A., Mertowska P., Grywalska E. (2023). How Do Microorganisms Influence the Development of Endometriosis? Participation of Genital, Intestinal and Oral Microbiota in Metabolic Regulation and Immunopathogenesis of Endometriosis. Int. J. Mol. Sci..

[110] Zhao C., Wei Z., Yang J., Zhang J., Yu C., Yang A., Zhang M., Zhang L., Wang Y., Mu X. (2020). Characterization of the Vaginal Microbiome in Women with Infertility and Its Potential Correlation with Hormone Stimulation during In Vitro Fertilization Surgery. mSystems.

[111] Carosso A., Revelli A., Gennarelli G., Canosa S., Cosma S., Borella F., Tancredi A., Paschero C., Boatti L., Zanotto E. (2020). Controlled ovarian stimulation and progesterone supplementation affect vaginal and endometrial microbiota in IVF cycles: A pilot study. J. Assist. Reprod. Genet..

[112] Park H., Yeo S., Ryu C.B., Huh C.S. (2024). A streamlined culturomics case study for the human gut microbiota research. Sci. Rep..

[113] Lagier J.C., Dubourg G., Million M., Cadoret F., Bilen M., Fenollar F., Levasseur A., Rolain J.M., Fournier P.E., Raoult D. (2018). Culturing the human microbiota and culturomics. Nat. Rev. Microbiol..

[114] Günther V., Allahqoli L., Watrowski R., Maass N., Ackermann J., von Otte S., Alkatout I. (2022). Vaginal Microbiome in Reproductive Medicine. Diagnostics.

[115] Li F., Liu J., Maldonado-Gómez M.X., Frese S.A., Gänzle M.G., Walter J. (2024). Highly accurate and sensitive absolute quantification of bacterial strains in human fecal samples. Microbiome.

[116] Quince C., Walker A.W., Simpson J.T., Loman N.J., Segata N. (2017). Shotgun metagenomics, from sampling to analysis. Nat. Biotechnol..

[117] Yuan S., Cohen D.B., Ravel J., Abdo Z., Forney L.J. (2012). Evaluation of methods for the extraction and purification of DNA from the human microbiome. PLoS ONE.

[118] Nowicki C., Ray L., Engen P., Madrigrano A., Witt T., Lad T., Cobleigh M., Mutlu E.A. (2023). Comparison of gut microbiome composition in colonic biopsies, endoscopically-collected and at-home-collected stool samples. Front. Microbiol..

[119] Zmora N., Zilberman-Schapira G., Suez J., Mor U., Dori-Bachash M., Bashiardes S., Kotler E., Zur M., Regev-Lehavi D., Brik R.B. (2018). Personalized Gut Mucosal Colonization Resistance to Empiric Probiotics Is Associated with Unique Host and Microbiome Features. Cell.

[120] van Rossen T.M., van den Hoek K., Groot T., Creemers R.H., van Doorn-Schepens M.L.M., van Andel E.M., Grasman M.E., de Boer N.K.H., Bouma G., Budding A.E. (2026). Comparison of microbiota profiles of fecal samples, rectal swabs and mucosal biopsies in patients with inflammatory bowel disease. Gut Microbes Rep..

[121] Mukhopadhya I., Martin J.C., Shaw S., McKinley A.J., Gratz S.W., Scott K.P. (2022). Comparison of microbial signatures between paired faecal and rectal biopsy samples from healthy volunteers using next-generation sequencing and culturomics. Microbiome.

[122] Peric A., Weiss J., Vulliemoz N., Baud D., Stojanov M. (2019). Bacterial Colonization of the Female Upper Genital Tract. Int. J. Mol. Sci..

[123] Zhou Z., Feng Y., Xie L., Ma S., Cai Z., Ma Y. (2024). Alterations in gut and genital microbiota associated with gynecological diseases: A systematic review and meta-analysis. Reprod. Biol. Endocrinol..

[124] Dourado E., Ferro M., Sousa Guerreiro C., Fonseca J.E. (2020). Diet as a Modulator of Intestinal Microbiota in Rheumatoid Arthritis. Nutrients.

[125] Moszak M., Szulińska M., Bogdański P. (2020). You Are What You Eat-The Relationship between Diet, Microbiota, and Metabolic Disorders—A Review. Nutrients.

[126] Min L., Ablitip A., Wang R., Luciana T., Wei M., Ma X. (2024). Effects of Exercise on Gut Microbiota of Adults: A Systematic Review and Meta-Analysis. Nutrients.

[127] Wang M.Y., Sang L.X., Sun S.Y. (2024). Gut microbiota and female health. World J. Gastroenterol..

[128] Gibson G.R., Hutkins R., Sanders M.E., Prescott S.L., Reimer R.A., Salminen S.J. (2017). Expert consensus document: The International Scientific Association for Probiotics and Prebiotics (ISAPP) consensus statement on the definition and scope of prebiotics. Nat. Rev. Gastroenterol. Hepatol..

[129] Fernández G., Santagada A.L., Vecchiarelli C., Vinderola G. (2025). Lactation, mastitis, and probiotics. Arch. Argent. Pediatr..

[130] Ramzan H., Bukhari D.A., Bibi Z., Arifullah, Isha, Nawaz A., Rehman A. (2025). Probiotic supplement for the treatment of polycystic ovarian syndrome. Pharmacol. Ther..

[131] Abbasi A., Aghebati-Maleki A., Yousefi M., Aghebati-Maleki L.J. (2021). Probiotic intervention asa potential therapeutic for managing gestational disorders and improving pregnancy outcomes. Reprod. Immunol..

[132] Zhang J., Sun Z., Jiang S., Bai X., Ma C., Peng Q., Chen K., Chang H., Fang T., Zhang H. (2019). Probiotic Bifidobacterium lactis V9 Regulates the Secretion of Sex Hormones in Polycystic Ovary Syndrome Patients through the Gut-Brain Axis. mSystems.

[133] Ngugi B.M., Hemmerling A., Bukusi E.A., Kikuvi G., Gikunju J., Shiboski S., Fredricks D.N., Cohen C.R. (2011). Effects of bacterial vaginosis-associated bacteria and sexual intercourse on vaginal colonization with the probiotic Lactobacillus crispatus CTV-05. Sex. Transm. Dis..

[134] Hemmerling A., Mitchell C.M., Demby S., Ghebremichael M., Elsherbini J., Xu J., Xulu N., Shih J., Dong K., Govender V. (2025). Effect of the vaginal live biotherapeutic LACTIN-V (Lactobacillus crispatus CTV-05) on vaginal microbiota and genital tract inflammation among women at high risk of HIV acquisition in South Africa: A phase 2, randomised, placebo-controlled trial. Lancet Microbe.

[135] Oerlemans E.F.M., Bellen G., Claes I., Henkens T., Allonsius C.N., Wittouck S., van den Broek M.F.L., Wuyts S., Kiekens F., Donders G.G.G. (2020). Impact of a lactobacilli-containing gel on vulvovaginal candidosis and the vaginal microbiome. Sci. Rep..

[136] Bradshaw C.S., Pirotta M., De Guingand D., Hocking J.S., Morton A.N., Garland S.M., Fehler G., Morrow A., Walker S., Vodstrcil L.A. (2012). Efficacy of oral metronidazole with vaginal clindamycin or vaginal probiotic for bacterial vaginosis: Randomised placebo-controlled double-blind trial. PLoS ONE.

[137] Zakaria I.A., Mohammed Zain N.A., Teik C.K., Abu M.A., Zainuddin A.A., Abdul Aziz N.H. (2024). The role of probiotics in improving menstrual health in women with primary dysmenorrhoea: A randomized, double-blind, placebo-controlled trial (the PERIOD study). Womens Health.

[138] Khodaverdi S., Mohammadbeigi R., Khaledi M., Mesdaghinia L., Sharifzadeh F., Nasiripour S. (2019). Beneficial Effects of Oral Lactobacillus on Pain Severity in Women Suffering from Endometriosis: A Pilot Placebo-Controlled Randomized Clinical Trial. Int. J. Fertil. Steril..

[139] Anania C., Matys V., Marra S., De Canditiis D., Olivero F., Carraro C. (2025). Effect of Supplementation with a Specific Probiotic (*Bifidobacterium bifidum* PRL2010) in Pregnancy for the Prevention of Atopic Dermatitis in Children: Preliminary Results of a Randomized Trial. Nutrients.

[140] Li Y.K., Xiao C.L., Ren H., Li W.R., Guo Z., Luo J.Q. (2024). Comparison of the effectiveness of probiotic supplementation in glucose metabolism, lipid profile, inflammation and oxidative stress in pregnant women. Food Funct..

[141] Ma G., Yan H., Tye K.D., Tang X., Luo H., Li Z., Xiao X. (2024). Effect of probiotic administration during pregnancy on the functional diversity of the gut microbiota in healthy pregnant women. Microbiol. Spectr..

[142] Luoto R., Kalliomäki M., Laitinen K., Isolauri E. (2010). The impact of perinatal probiotic intervention on the development of overweight and obesity: Follow-up study from birth to 10 years. Int. J. Obes..

[143] Mehlferber E.C., Arnault G., Joshi B., Partida-Martinez L.P., Patras K.A., Simonin M., Koskella B. (2024). A cross-systems primer for synthetic microbial communities. Nat. Microbiol..

[144] Li Y., Zhu W., Jiang Y., Lessing D.J., Chu W. (2023). Synthetic bacterial consortia transplantation for the treatment of Gardnerella vaginalis-induced bacterial vaginosis in mice. Microbiome.

[145] De Roy K., Marzorati M., Van den Abbeele P., Van de Wiele T., Boon N. (2014). Synthetic microbial ecosystems: An exciting tool to understand and apply microbial communities. Environ. Microbiol..

[146] Vinderola G., Sanders M.E., Salminen S., Szajewska H. (2022). Postbiotics: The concept and their use in healthy populations. Front. Nutr..

[147] Zhang Y., Pu F., Cheng R., Guo J., Shen X., Wang S. (2020). Effect of heat-inactivated *Lactobacillus paracasei* N1115 on microbiota and gut-brain axis related molecules. Biosci. Microbiota Food Health.

[148] Zhu C., Gong L., Huang K., Li F., Tong D., Zhang H. (2020). Effect of Heat-Inactivated Compound Probiotics on Growth Performance, Plasma Biochemical Indices, and Cecal Microbiome in Yellow-Feathered Broilers. Front. Microbiol..

[149] Toca M.D.C., Burgos F., Tabacco O., Vinderola G. (2024). Postbiotics: A new member in the biotics family. Arch. Argent. Pediatr..

[150] Yadegar A., Bar-Yoseph H., Monaghan T.M., Pakpour S., Severino A., Kuijper E.J., Smits W.K., Terveer E.M., Neupane S., Nabavi-Rad A. (2024). Fecal microbiota transplantation: Current challenges and future landscapes. Clin. Microbiol. Rev..

[151] Lev-Sagie A., Goldman-Wohl D., Cohen Y., Dori-Bachash M., Leshem A., Mor U., Strahilevitz J., Moses A.E., Shapiro H., Yagel S. (2019). Vaginal microbiome transplantation in women with intractable bacterial vaginosis. Nat. Med..

[152] Huang L., Liu B., Liu Z., Feng W., Liu M., Wang Y., Peng D., Fu X., Zhu H., Cui Z. (2021). Gut Microbiota Exceeds Cervical Microbiota for Early Diagnosis of Endometriosis. Front. Cell. Infect. Microbiol..

[153] Guo Y., Qi Y., Yang X., Zhao L., Wen S., Liu Y., Tang L. (2016). Association between Polycystic Ovary Syndrome and Gut Microbiota. PLoS ONE.

[154] Chadchan S.B., Cheng M., Parnell L.A., Yin Y., Schriefer A., Mysorekar I.U. (2019). Antibiotic therapy with metronidazole reduces endometriosis disease progression in mice: A potential role for gut microbiota. Hum. Reprod..

[155] Wrønding T., Vomstein K., Bosma E.F., Mortensen B., Westh H., Heintz J.E., Mollerup S., Petersen A.M., Ensign L.M., DeLong K. (2023). Antibiotic-free vaginal microbiota transplant with donor engraftment, dysbiosis resolution and live birth after recurrent pregnancy loss: A proof of concept case study. eClinicalMedicine.

[156] Sofi H.S., Abdal-Hay A., Ivanovski S., Zhang Y.S., Sheikh F.A. (2020). Electrospun nanofibers for the delivery of active drugs through nasal, oral and vaginal mucosa: Current status and future perspectives. Mater. Sci. Eng. C Mater. Biol. Appl..

[157] Yamamoto H.S., Xu Q., Fichorova R.N. (2013). Homeostatic properties of Lactobacillus jensenii engineered as a live vaginal anti-HIV microbicide. BMC Microbiol..

[158] ClinicalTrials.gov. A Phase 1 Trial to Assess the Safety of MucoCept-CVN (Identifier: NCT07181486). NCT07181486.

[159] Steidler L., Hans W., Schotte L., Neirynck S., Obermeier F., Falk W., Fiers W., Remaut E. (2000). Treatment of murine colitis by Lactococcus lactis secreting interleukin-10. Science.

[160] Braat H., Rottiers P., Hommes D.W., Huyghebaert N., Remaut E., Remon J.P., van Deventer S.J., Neirynck S., Peppelenbosch M.P., Steidler L. (2006). A phase I trial with transgenic bacteria expressing interleukin-10 in Crohn’s disease. Clin. Gastroenterol. Hepatol..

[161] He L., Yang H., Tang J., Liu Z., Chen Y., Lu B., He H., Tang S., Sun Y., Liu F. (2019). Intestinal probiotics E. *coli Nissle* 1917 as a targeted vehicle for delivery of p53 and Tum-5 to solid tumors for cancer therapy. J. Biol. Eng..

[162] Gurbatri C.R., Lia I., Vincent R., Coker C., Castro S., Treuting P.M., Hinchliffe T.E., Arpaia N., Danino T. (2020). Engineered probiotics for local tumor delivery of checkpoint blockade nanobodies. Sci. Transl. Med..

[163] Bermúdez-Humarán L.G., Langella P., Cortes-Perez N.G., Gruss A., Tamez-Guerra R.S., Oliveira S.C., Saucedo-Cardenas O., Montes de Oca-Luna R., Le Loir Y. (2003). Intranasal immunization with recombinant Lactococcus lactis secreting murine interleukin-12 enhances antigen-specific Th1 cytokine production. Infect. Immun..

[164] Dai M., Liu S., Cai R., Li D., Wen Y., Jiang J., Hao N., Wang Q., Gao X. (2025). Engineering *Escherichia coli* nissle 1917 with lactonase ZHD101 alleviates zearalenone-induced intestinal disruption and reproductive toxicity in rat. World J. Microbiol. Biotechnol..

[165] Liu S., Zhang Y., Ma X., Zhan C., Ding N., Shi M., Zhang W., Yang S. (2024). Protective effects of engineered Lactobacillus crispatus strains expressing G-CSF on thin endometrium of mice. Hum. Reprod..

[166] Petrova M.I., van den Broek M.F.L., Spacova I., Verhoeven T.L.A., Balzarini J., Vanderleyden J., Schols D., Lebeer S. (2018). Engineering Lactobacillus rhamnosus GG and GR-1 to express HIV-inhibiting griffithsin. Int. J. Antimicrob. Agents.

[167] Liu X., Lagenaur L.A., Simpson D.A., Essenmacher K.P., Frazier-Parker C.L., Liu Y., Tsai D., Rao S.S., Hamer D.H., Parks T.P. (2006). Engineered vaginal lactobacillus strain for mucosal delivery of the human immunodeficiency virus inhibitor cyanovirin-N. Antimicrob. Agents Chemother..

[168] Landlinger C., Tisakova L., Oberbauer V., Schwebs T., Muhammad A., Latka A., Van Simaey L., Vaneechoutte M., Guschin A., Resch G. (2021). Engineered Phage Endolysin Eliminates Gardnerella Biofilm without Damaging Beneficial Bacteria in Bacterial Vaginosis Ex Vivo. Pathogens.

[169] Citorik R.J., Mimee M., Lu T.K. (2014). Sequence-specific antimicrobials using efficiently delivered RNA-guided nucleases. Nat. Biotechnol..

[170] Neil K., Allard N., Roy P., Grenier F., Menendez A., Burrus V., Rodrigue S. (2021). High-efficiency delivery of CRISPR-Cas9 by engineered probiotics enables precise microbiome editing. Mol. Syst. Biol..

[171] Ramachandran G., Bikard D. (2019). Editing the microbiome the CRISPR way. Philos. Trans. R Soc. Lond. B Biol. Sci..

[172] Chieng W.K., Abdul Jalal M.I., Bedi J.S., Zainuddin A.A., Mokhtar M.H., Abu M.A., Chew K.T., Nur Azurah A.G. (2022). Probiotics, a promising therapy to reduce the recurrence of bacterial vaginosis in women? A systematic review and meta-analysis of randomized controlled trials. Front. Nutr..

[173] Muñoz-Barreno A., Cabezas-Mera F., Tejera E., Machado A. (2021). Comparative Effectiveness of Treatments for Bacterial Vaginosis: A Network Meta-Analysis. Antibiotics.

[174] Mahdizade Ari M., Dadgar L., Elahi Z., Ghanavati R., Taheri B. (2024). Genetically Engineered Microorganisms and Their Impact on Human Health. Int. J. Clin. Pract..

[175] Wei G., Liu Q., Wang X., Zhou Z., Zhao X., Zhou W., Liu W., Zhang Y., Liu S., Zhu C. (2023). A probiotic nanozyme hydrogel regulates vaginal microenvironment for Candida vaginitis therapy. Sci. Adv..

[176] Chumduri C., Turco M.Y. (2021). Organoids of the female reproductive tract. J. Mol. Med..

[177] Gargus E.S., Rogers H.B., McKinnon K.E., Edmonds M.E., Woodruff T.K. (2020). Engineered reproductive tissues. Nat. Biomed. Eng..

[178] Charbonneau M.R., Isabella V.M., Li N., Kurtz C.B. (2020). Developing a new class of engineered live bacterial therapeutics to treat human diseases. Nat. Commun..

[179] Altmäe S., Franasiak J.M., Mändar R. (2019). The seminal microbiome in health and disease. Nat. Rev. Urol..

